# Integrating Artificial Intelligence and Bioinformatics Methods to Identify Disruptive STAT1 Variants Impacting Protein Stability and Function

**DOI:** 10.3390/genes16030303

**Published:** 2025-03-01

**Authors:** Ebtihal Kamal, Lamis A. Kaddam, Mehad Ahmed, Abdulaziz Alabdulkarim

**Affiliations:** 1 Department of Basic Medical Sciences, College of Medicine, Prince Sattam bin Abdulaziz University, Al Kharj 16278, Saudi Arabia; 2 Department of Physiology, Faculty of Medicine, King Abdul-Aziz University, Rabigh 25724, Saudi Arabia; 3 Plastic Surgery, Department of Surgery, College of Medicine, Prince Sattam bin Abdulaziz University, Al Kharj 16278, Saudi Arabia

**Keywords:** in silico, single-nucleotide polymorphism, *STAT1*, autoimmunity, signaling pathway

## Abstract

**Background:** The Signal Transducer and Activator of Transcription 1 (*STAT1*) gene is an essential component of the JAK-STAT signaling pathway. This pathway plays a pivotal role in the regulation of different cellular processes, including immune responses, cell growth, and apoptosis. Mutations in the *STAT1* gene contribute to a variety of immune system dysfunctions. **Objectives:** We aim to identify disease-susceptible single-nucleotide polymorphisms (SNPs) in *STAT1* gene and predict structural changes associated with the mutations that disrupt normal protein–protein interactions using different computational algorithms. **Methods:** Several in silico tools, such as SIFT, Polyphen v2, PROVEAN, SNAP2, PhD-SNP, SNPs&GO, Pmut, and PANTHER, were used to determine the deleterious nsSNPs of the *STAT1*. Further, we evaluated the potentially deleterious SNPs for their effect on protein stability using I-Mutant, MUpro, and DDMUT. Additionally, we predicted the functional and structural effects of the nsSNPs using MutPred. We used Alpha-Missense to predict missense variant pathogenicity. Moreover, we predicted the 3D structure of STAT1 using an artificial intelligence system, alphafold, and the visualization of the 3D structures of the wild-type amino acids and the mutant residues was performed using ChimeraX 1.9 software. Furthermore, we analyzed the structural and conformational variations that have resulted from SNPs using Project Hope, while changes in the biological interactions between wild type, mutant amino acids, and neighborhood residues was studied using DDMUT. Conservational analysis and surface accessibility prediction of STAT1 was performed using ConSurf. We predicted the protein–protein interaction using STRING database. **Results:** In the current study, we identified six deleterious nsSNPs (R602W, I648T, V642D, L600P, I578N, and W504C) and their effect on protein structure, function, and stability. **Conclusions:** These findings highlight the potential of approaches to pinpoint pathogenic SNPs, providing a time- and cost-effective alternative to experimental approaches. To the best of our knowledge, this is the first comprehensive study in which we analyze *STAT1* gene variants using both bioinformatics and artificial-intelligence-based model tools.

## 1. Introduction

Signal Transducer and Activator of Transcription (STAT) proteins are a family of transcription factors latently present in the cytoplasm and participate in a variety of cellular events following cytokines and growth factors signaling [1,2]. STAT proteins are involved in intracellular signaling downstream of the type I and type II cytokine receptors. Upon activation, translocation to the nucleus, binding to their specific promoter regions of target genes and regulation of their transcription subsequently takes place [3,4]. Seven proteins have been identified (STAT1, -2, -3, -4, -5a, -5b, and -6) and share a common structure consisting of an SH2 domain that mediates STAT interactions through homo- or heterodimers, a coiled-coil domain, which is important for dimer nuclear localization, a DNA-binding domain, which leads to target gene transcription, and a transactivation domain [5,6].

The Signal Transducer and Activator of Transcription 1 (*STAT1*) gene is composed of 25 exons and 7 domains, located on chromosome 2q32.2 [7,8,9]. STAT1 is an essential mediator of the JAK-STAT signaling pathway in response to interferons [8,10,11,12]. It plays a crucial role in the biological immune response against intracellular mycobacterial infection as well as viral infections [8,13,14]. Upon type I IFN-gamma (IFN-γ) binding to cell surface receptors, there is a signaling pathway through protein kinases then activation of Jak kinases (TYK2 and JAK1) with tyrosine phosphorylation of STAT1, dimerization of phosphorylated STAT1, and association with ISGF3G/IRF-9 forming ISGF3 transcription factor [15]. ISGF3 enters the nucleus and binds to the IFN-stimulated response element (ISRE) to activate the transcription of IFN-stimulated genes (ISG), which bring the cell into an antiviral state [16]. Moreover, in response to type II IFN, STAT1 is tyrosine- and serine-phosphorylated; it then forms a homodimer termed IFN-gamma-activated factor (GAF) [17] that migrates into the nucleus and binds to the IFN-gamma-activated sequence (GAS) to drive the expression of the target genes, inducing a cellular antiviral state [18].

Genetic variants within *STAT1* gene lead to loss-of-function (LOF) and gain-of-function (GOF) phenotypes, with a wide range of clinical presentations, including autoimmunity and life-threatening mycobacterial, severe viral, and bacterial infections [19,20,21]. *STAT1* amorphic alleles cause severe viral and bacterial infections, while hypomorphic alleles cause mild disseminated mycobacterial disease [22]. Moreover, hypermorphic mutations are responsible for a variety of clinical presentations such as chronic mucocutaneous candidiasis (CMC), arterial aneurysms, autoimmunity, and squamous cell cancers [23]. *STAT1* gain-of-function (GOF) mutation, mostly located at coiled-coil (CCD) and DNA-binding domains (DBD) causing hyper-phosphorylation of STAT1 protein, thus enhanced STAT1-dependent responses to interferons (IFNs) and IL-27, with sequential impairment of Th17 cell development [24,25,26]. GOF mutation is associated with chronic mucocutaneous candidiasis [10,27,28], while patients with LOF mutations display an increased susceptibility to intracellular bacteria, including a Mendelian susceptibility to mycobacterial disease (MSMD) [10,22].

Single-nucleotide polymorphisms (SNPs) constitute a common form of genetic variation in humans [29]. The nonsynonymous SNPs (nsSNPs) cause alteration in the amino acid residues because of variation in the sequence of DNA at a single position of a nucleotide (A, T, C, or G), which contributes to the functional diversity of the related proteins [30,31,32].

Recently, bioinformatics tools have played a significant role in the prediction of damaging SNPs and their relationship with diseases [33]. The influence of *STAT1* nsSNPs on protein structure and function has not been thoroughly investigated, despite their potential importance; this indicates a substantial scientific gap. Nonetheless, limited published articles have systematically examined STAT1 SNPs by bioinformatics approaches.

The objective of this study is to define the structural and functional characterization of the most pathogenic variations of the *STAT1* gene. We performed a comprehensive *STAT1*-SNPs analysis using bioinformatics prediction tools combined with artificial intelligence models to identify the pathogenic and deleterious SNPs, providing novel insights into their involvement in immune dysregulation and establishing a foundation for subsequent functional and clinical research.

## 2. Materials and Method

**Figure 1:**
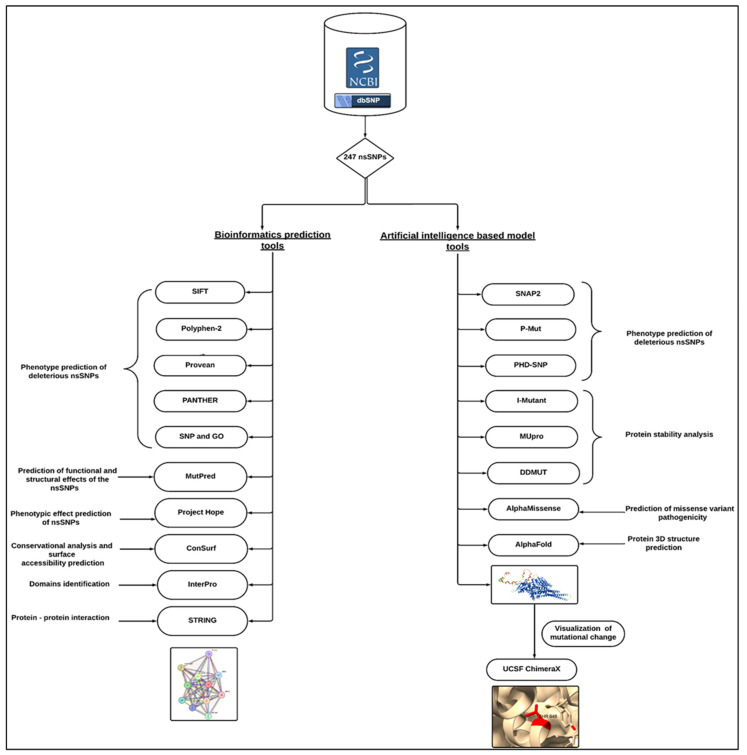
Workflow of the analysis.

An overview of the complete methodological approach is shown in Figure 1.

### 2.1. Data Retrieval

We gathered the data for the human *STAT1* gene from the National Center for Biological Information (NCBI) website (https://www.ncbi.nlm.nih.gov/) (accessed on 20 April 2024). While the SNP information (SNP ID) of the *STAT1* gene was obtained from the NCBI dbSNP (https://www.ncbi.nlm.nih.gov/gene/?term=STAT1, accessed on 20 April 2024), the protein ID and its sequence were extracted from UniProtKB in Swiss-Prot databases with the accession number P42224 (https://www.expasy.org/search/uniprot, accessed on 20 April 2024) [34].

### 2.2. Phenotype Prediction of Deleterious ns SNPs

We predicted the deleterious nsSNPs by using eight different tools. Sorting Intolerant from Tolerant (SIFT) (http://sift.bii.a-star.edu.sg/, accessed on 20 April 2024) predicts whether the replacement of an amino acid alters protein function. We downloaded nsSNP IDs from the online databases of NCBI and then uploaded them to SIFT. Results were documented as damaging (deleterious) or benign (tolerated), depending on the cutoff value of 0.05, as values less than or equal to (0.0–0.04) were predicted to be damaging or intolerant, while (0.05_1) is benign or tolerated [35].

Polyphen-2 (http://genetics.bwh.harvard.edu/pph2/, accessed on 20 April 2024) analyzes multiple sequence alignments and the protein’s three-dimensional structure, then predicts the possible impact of amino acid substitutions on the stability and function of human proteins using structural and comparative evolutionary considerations. The prediction outcomes are classified as probably damaging, possibly damaging, or benign based on the position-specific independent counts value (PSIC), which ranges from 0 to 1. Values near zero are regarded as benign, while values near one are considered probably damaging [36].

Provean (https://www.jcvi.org/research/provean/, accessed on 20 April 2024) is a software tool that predicts whether an amino acid substitution or indel has an impact on the biological function of a protein. Variants with a score equal to or below −2.5 are considered deleterious, while variants with a score above −2.5 are neutral [37].

SNAP2 (https://rostlab.org/services/snap2web/, accessed on 20 April 2024) is a trained classifier that uses the “neural network” machine learning tool to predict the functional effects of mutations by utilizing several sequence and variant properties to discriminate between effect and neutral variants/nonsynonymous SNPs [38].

PHD-SNP (https://snps.biofold.org/phd-snp/phd-snp.html, accessed on 20 April 2024) uses a support vector machine (SVM)-based method trained to determine disease-associated nsSNPs using sequence information. PHD-SNP classifies mutations either to be disease-related (disease) or a neutral polymorphism [39].

SNP and GO (https://snps-and-go.biocomp.unibo.it/snps-and-go/, accessed on 20 April 2024) is a server for the prediction of single-point protein mutations likely to be involved in the development of diseases in humans [40].

P-Mut is a web-based tool for the annotation of pathological variants on proteins. It allows fast and accurate prediction of the pathological properties of single-point amino acid mutations based on the use of a neural network. It is available at (http://mmb.irbbarcelona.org/PMut, accessed on 20 April 2024) [41].

Protein Analysis through Evolutionary Relationships (PANTHER) (http://pantherdb.org/, accessed on 20 April 2024) uses a position-specific evolutionary preservation (PSEP) score to measure the length of time (in millions of years), with <200 my “probably benign”, <450 my “possibly damaging”, and 450 my “probably damaging” [42].

### 2.3. Predicting Functional and Structural Effects of the nsSNP

MutPred v1.2 (http://mutpred.mutdb.org/, accessed on 20 April 2024) is used for sorting disease-associated or neutral amino acid substitutions in humans. MutPred is an efficient web-based application tool that screens amino acid substitutions and predicts the molecular base of the disease [43].

### 2.4. Protein Stability Analysis of Predicted STAT1 nsSNPs

I-Mutant 3.0 is available at (https://gpcr2.biocomp.unibo.it/cgi/predictors/I-Mutant3.0/I-Mutant3.0.cgi, accessed on 20 April 2024); it is a neural-network-based tool for routinely analyzing protein stability and change while taking single-site mutations into consideration [44]. The FASTA sequence of proteins retrieved from UniProt is used as an input to predict the mutational effect on protein stability.

MUpro, a group of machine learning methods, predicts the effects of single amino acid substitutions on protein stability [45]. It uses both support vector machines and neural networks; the output is either increased or decreased stability [45]. MUpro also interprets the result based on Gibbs free energy (ΔΔG), with a confidence score between −1 and 11. It is available at http://mupro.proteomics.ics.uci.edu, accessed on 20 April 2024.

DDMUT (https://biosig.lab.uq.edu.au/ddmut/, accessed on 20 April 2024) is a fast and accurate network using deep learning models to predict changes in Gibbs free energy (ΔΔG) upon single- and multiple-point mutations [46]. DDMut achieved a Pearson’s correlation of up to 0.70 (RMSE: 1.37 kcal/mol) on predicting single-point mutations on cross-validation and 0.74 (RMSE: 1.67 kcal/mol) on multiple mutations.

### 2.5. Prediction of Missense Variant Pathogenicity

Alpha Missense is an adaptation of alphafold fine-tuned on human and primate variant population frequency databases to predict missense variant pathogenicity. It works by combining structural context and evolutionary conservation. This model achieves state-of-the-art results across a wide range of genetic and experimental benchmarks, all without explicitly training on such data [47].

### 2.6. Three-Dimensional Structure Prediction and Visualization

We predicted the 3D structure using an artificial intelligence system, AlphaFold (https://alphafold.ebi.ac.uk, accessed on 20 April 2024). Alphafold is an artificial intelligence system developed by google DeepMind. It predicts a protein’s 3D structure from its amino acid sequence. It can predict protein structures computationally with high accuracy [48]. We used the UniProt sequence of the STAT1 protein as an input to obtain the alphafold model.

UCSF ChimeraX 1.9 is a robust application that enables interactive viewing and analysis of various molecular structures and related data, including density maps, sequence alignments, and supramolecular assemblies [49]. It allows the mapping and visualization of amino acid substitutions. Chimera X is available at https://www.rbvi.ucsf.edu/chimerax/, accessed on 20 April 2024.

### 2.7. Phenotypic Effects Prediction

Project Hope (version 1.0) is an online web server used to analyze the structural and conformational variations that have resulted from single amino acid substitutions [50]. We uploaded STAT1 protein sequence, wild-type amino acids, and mutants. The results provided describe the change in the physiochemical properties of the amino acid in the given SNPs. It is available at (https://www3.cmbi.umcn.nl/hope/method/, accessed on 20 April 2024).

DDMUT can also detect changes in the biological interactions between wild-type amino acids and neighborhood residues in comparison with mutant residues [46].

### 2.8. Conservational Analysis and Surface Accessibility Prediction of STAT1

The ConSurf bioinformatics tool (https://consurf.tau.ac.il, accessed on 20 April 2024) was used to study the evolutionary conservation of nsSNP positions in a protein sequence [51]. We submitted the FASTA sequence of the STAT1 protein to the server, and we screened out the highly conserved residues, exposed and buried residues.

### 2.9. Identification of nsSNPs in STAT1 Protein Domains

We submitted the FASTA sequence of the STAT1 protein to the InterPro server (https://www.ebi.ac.uk/interpro, accessed on 20 April 2024). It predicts protein families and conserved domains, and then we manually pinpointed the positions of nsSNPs within these domains [52].

### 2.10. Prediction of Protein–Protein Interactions

A precomputed database, STRING (https://string-db.org/, accessed on 20 April 2024), is used to determine protein–protein interactions to understand the function, structure, molecular action, and regulation of the protein [53]. We submitted the protein sequence as an input query.

## 3. Results

### 3.1. Distribution of STAT1 Gene SNP Datasets

**Figure 2:**
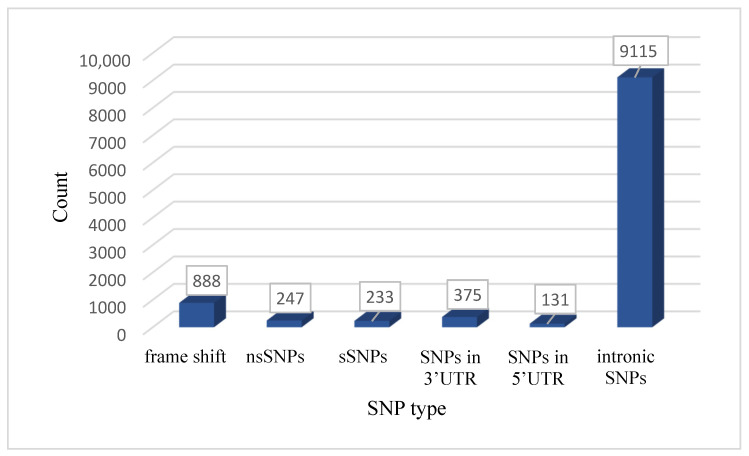
Shows the distribution of the SNPs in *STAT1* gene.

The total number of SNPs was 10,989. There were 888 frame shift mutations; 480 SNPs located in the coding region, of which 247 were nsSNPs and 233 were synonymous SNPs (sSNPs), while 9.621 SNPs were in noncoding regions, of which 375 occurred in the 3′UTR, 131 in the 5′UTR region, and the rest (9115) were in the intronic region, as shown in Figure 2. We chose nonsynonymous coding SNPs for our investigation.

### 3.2. Identification of Deleterious Missense Mutation

All 247 nsSNPs were retrieved and subjected to pathogenicity prediction web servers. Sixty-four nsSNPs were found to be deleterious by SIFT and were further subjected to crosschecking by using three different tools (Poly-Phen-2, PROVEAN, and SNAP2).

The shortlisted 33 nsSNPs passed the first four tools, presented in Table 1, then were submitted to another set of four tools: P Mut, PhD-SNP, SNPs and GO, and PANTHER. In total, 29 SNPs out of the 33 predicted by the first set of tools are disease-causing by P mut, 21 out of 33 are disease-causing by Panther, 20 are disease-causing by PhD-SNP, and 14 out of 33 by SNP and GO. A final nine nsSNPs passed all eight tools shown in Table 2. We further analyzed the final set of SNPs for the functional and structural modifications.

### 3.3. MutPred Prediction for Functional and Structural Modifications

We submitted the shortlisted nine nsSNPs to the MutPred server, along with the resultant probability scores and their *p* values in Table 3. The structural and functional alterations predicted include loss of disorder, catalytic residue, glycosylation, gain of phosphorylation, solvent accessibility, ubiquitination, and molecular recognition features (MoRF) binding. According to these predictions, several nsSNPs might be the reason behind any possible structural and functional modifications of STAT1 protein.

### 3.4. Prediction of Change in STAT1 Stability Due to Mutation

We used I mutant, MUpro, and DDMUT servers to predict the effect of the nsSNPs on protein stability. The result revealed that six variants destabilized the STAT 1 protein, namely (I648T) rs759271255, (V642D) rs752542806, (R602W) rs 1209841496, (L600P) rs137852678, (I578N) rs767475430, and (W504C) rs916580554. The results are presented in Table 4.

### 3.5. Pathogenicity Prediction Results

**Figure 3:**
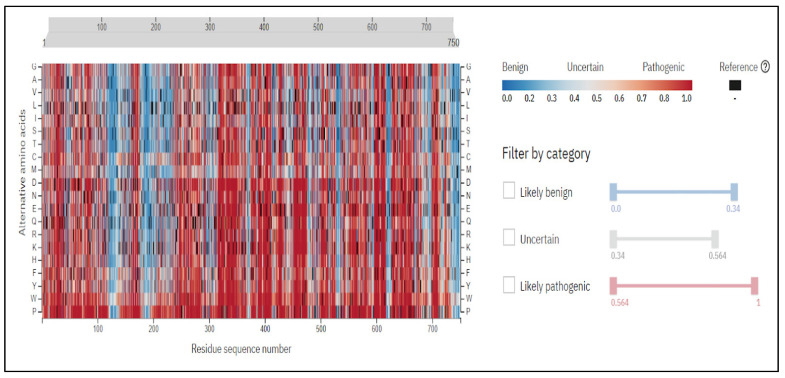
Heat map generated by Alpha-Missense shows the variations in *STAT1* gene.

We analyzed STAT1 nsSNP by Alpha-Missense, and we found that all the pathogenic nsSNPs that were predicted by the previous tools were also classified as pathogenic in Alpha-Missense, presented in Table 5. The heat map represented the mutations in STAT1, as shown in Figure 3.

### 3.6. The Conservational Status and Surface Accessibility Analysis of STAT1 Protein

Highly conserved residues are most likely to be involved in proteins’ structural integrity and functions. We evaluated the conservational profile for the STAT1 protein. The ConSurf algorithm represented the structural and functional conservation levels of all the amino acid residues of the STAT1 protein. Four SNPs (I648T, L600P, W504C, and I578N) are predicted to be located in a conserved region. L600P and I578N are predicted to be structural residues (highly conserved and buried). V642D is predicted as buried, and R602W is predicted as a functional residue (highly conserved and exposed), presented in Table 6.

### 3.7. Three-Dimensional Structure Prediction by AlphaFold and SNP Visualization by ChimeraX

**Figure 4:**
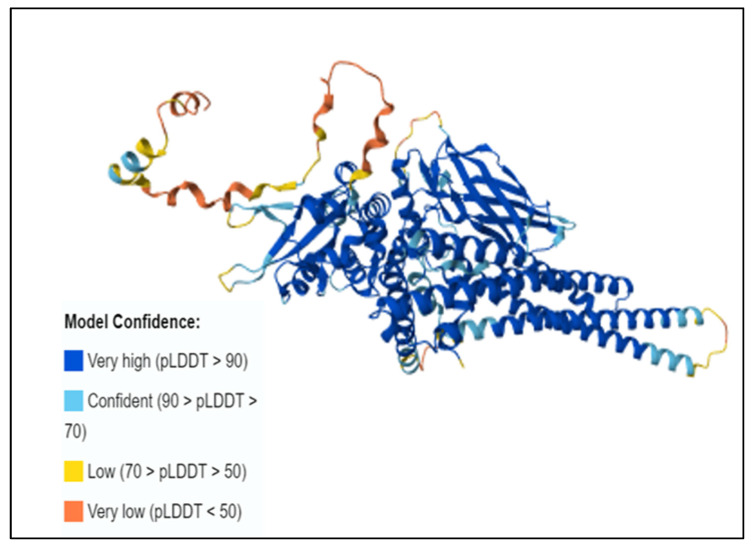
Protein 3D structure of human STAT1 predicted by AlphaFold2.

An individual residue confidence score (pLDDT) between 0 and 100 is generated by the AlphaFold algorithm. Alphafold produces a per residue confidence score (pLDDT) 1–100. Regions with low pLDDT may be unstructured in isolation. The majority of the 3D structural region corresponds to alpha-helical domains and has extremely high confidence (pLDDT > 90). The remaining components of the model are depicted as unresolved loops with low (70 > pLDDT > 50) and extremely low (pLDDT > 50) scores, as in Figure 4.

**Figure 5:**
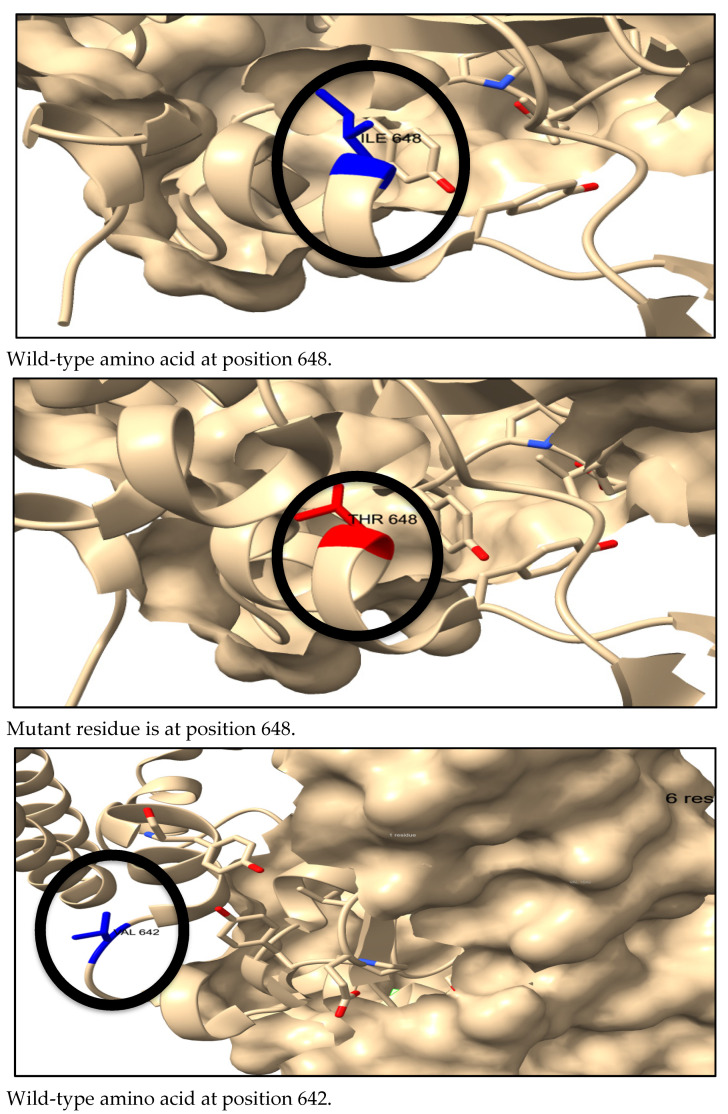

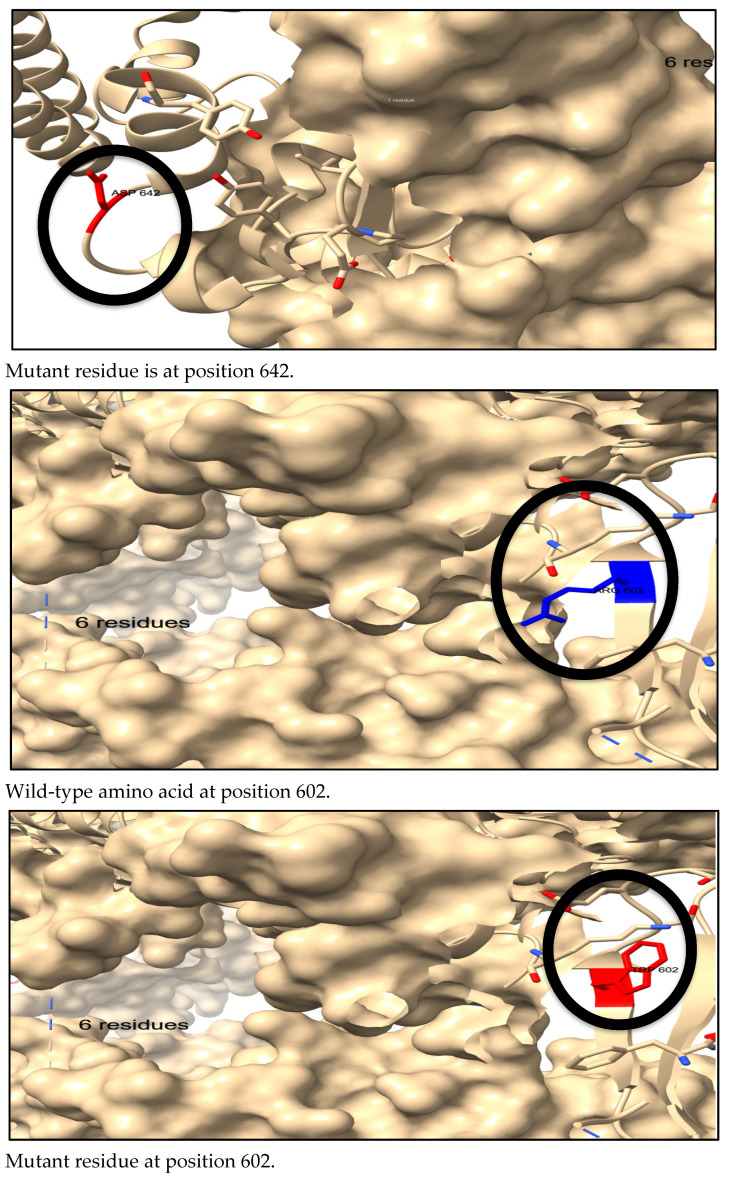

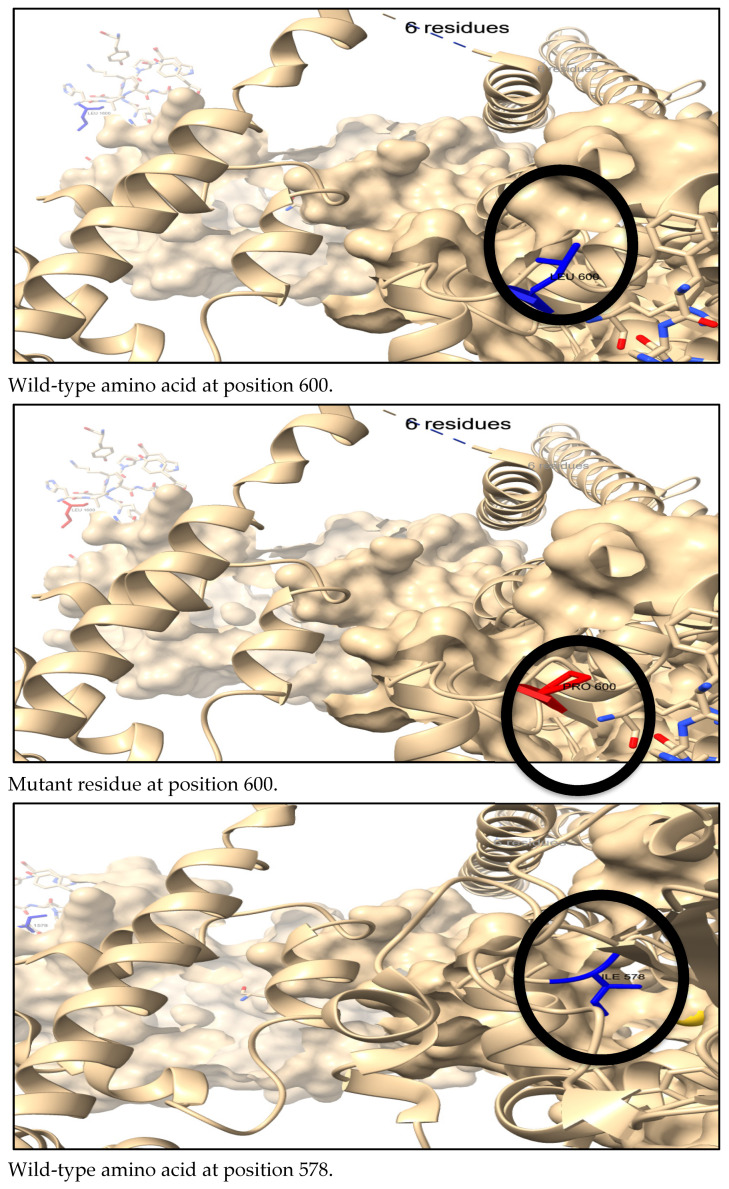

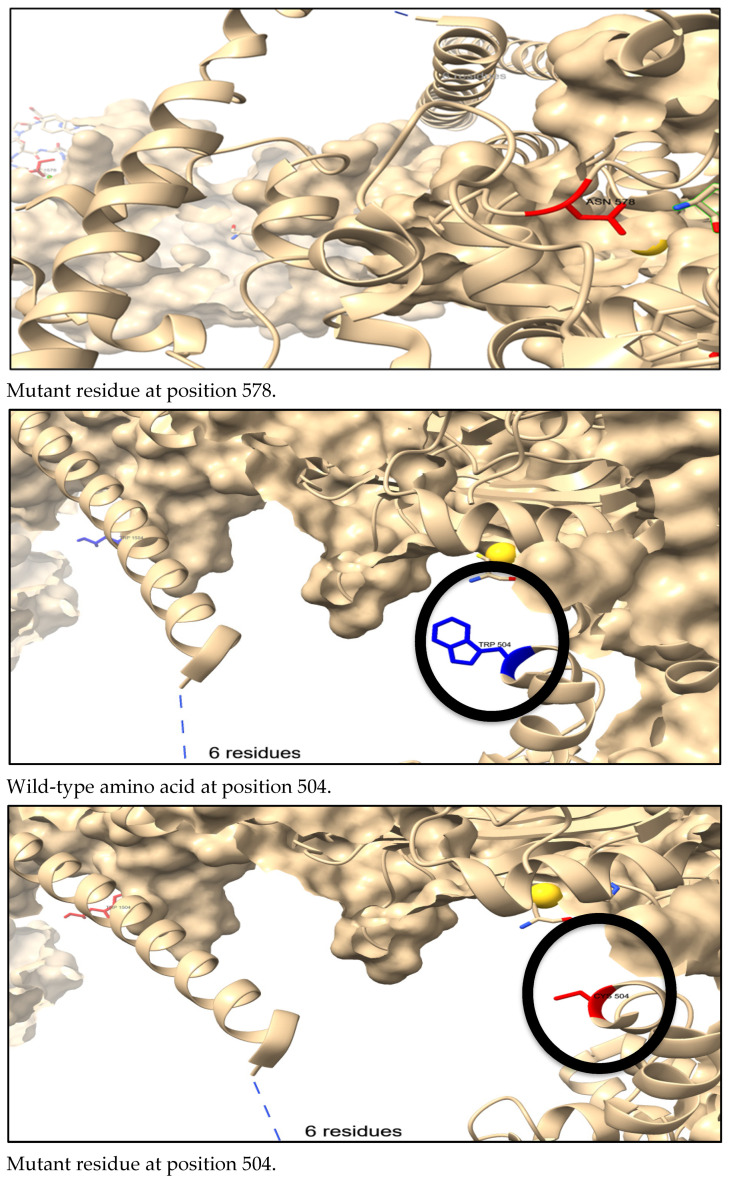
Effect of the six most deleterious nsSNPs on the STAT1 protein structure. ChimeraX software was used to visualize the 3D structure of the wild-type (blue), mutant residues (red) and gold ion (yellow).

We used ChimeraX to visualize the 3D structures of the wild-type amino acids in blue and the mutant residues in red, as shown in Figure 5.

### 3.8. The Physical Outcome of Predicted SNPs

We examined the impact of the generated damaging SNPs on the three-dimensional structure of STAT1 using the HOPE server. The server predicted that all the mutated amino acids were different in size; one had a different charge, and six had different hydrophobicity. The results are in Table 7.

**Figure 6:**
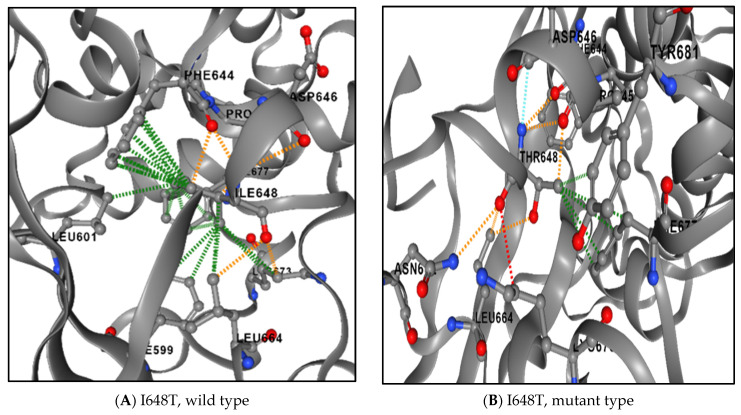

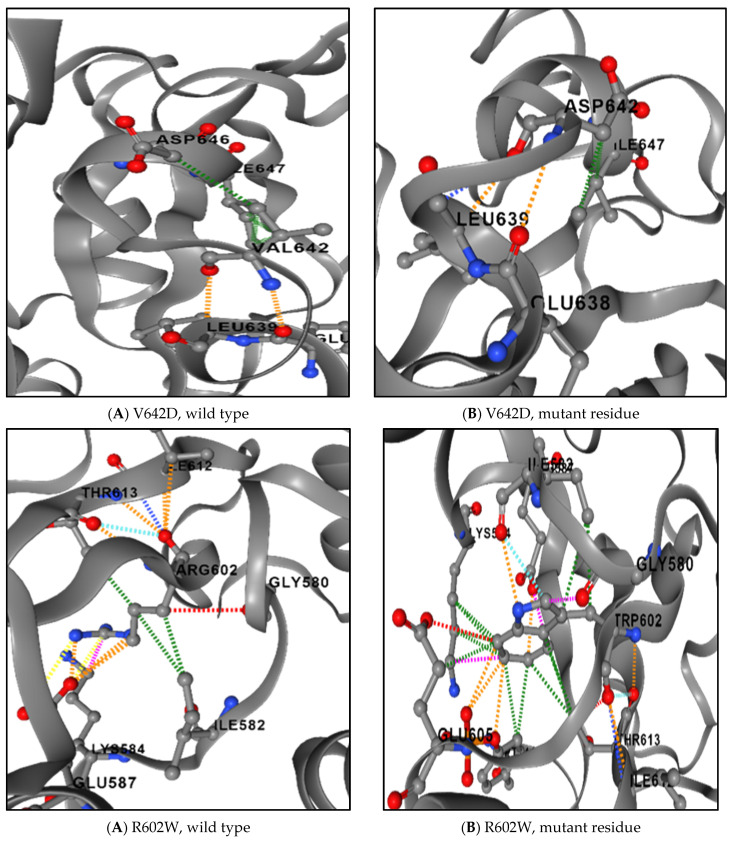

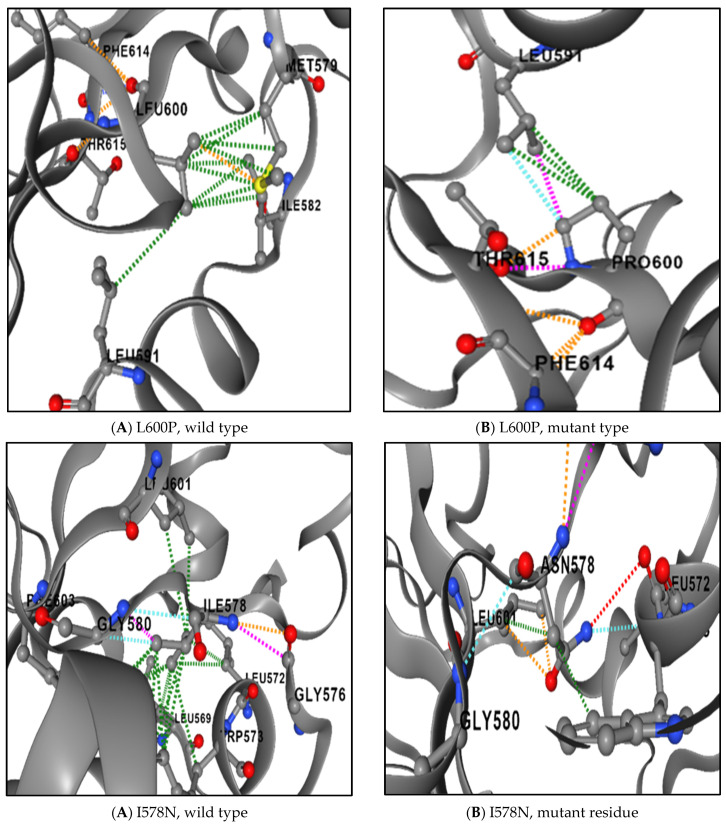

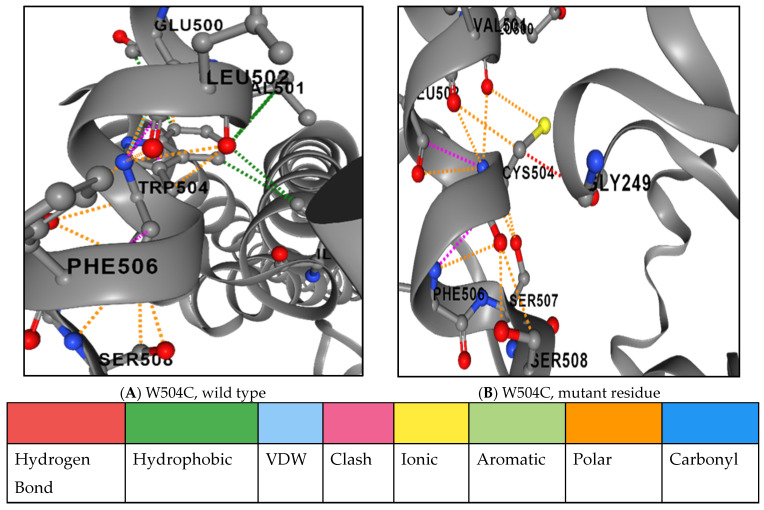
Difference in ionic interactions between the wild-type (**A**) and mutant residues (**B**).

Loss of the interactions between the wild-type amino acid and other amino acids in the protein and/or development of new interactions or bonds between the mutant residue of the protein and the other amino acids in the protein were predicted by DDMUT, as presented in Figure 6.

### 3.9. Domain Identification of the STAT1 Protein by the InterPro Server

The InterPro tool predicted the domain regions of the STAT1 protein. The STAT1, SH2 domain (a phosphotyrosine binding pocket) at position (557–707), STAT transcription factor, DNA binding domain at (323–458), and STAT1_TAZ2-binding domain (715–739) are conserved sites. Src homology 2 (SH2) domain profile (573–670), SH2 domain (578–638), STAT1 transcription factor, all alpha domain (144–305), and STAT transcription factor protein interaction (2–12) are as in Table 8.

### 3.10. STAT1–Protein Interaction

**Figure 7:**
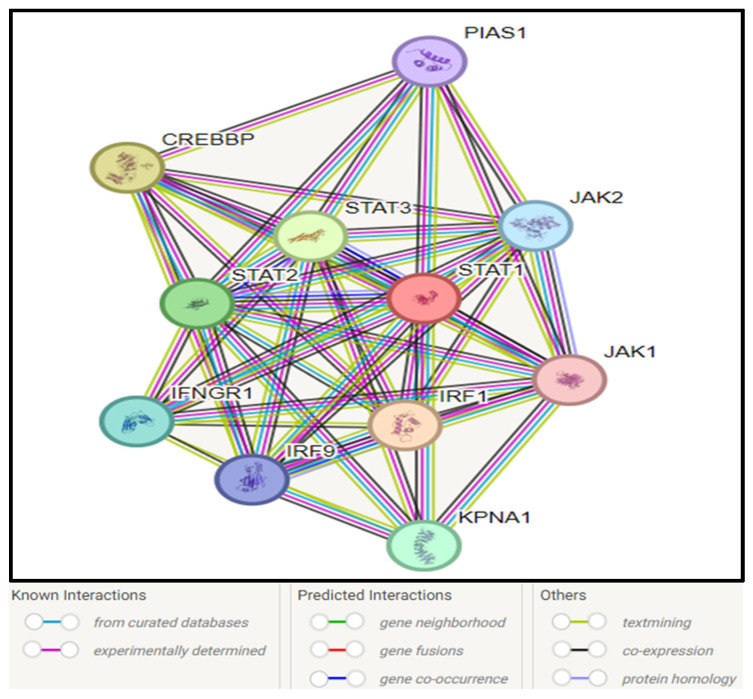
STAT1–protein interactions by STRING database.

Analysis of protein–protein interaction using the STRING network revealed that STAT1 interacts with 10 proteins, which include other proteins of the same STAT family (STAT2 and STAT3), proteins of the JAK family (JAK1 and JAK2), IFR1, IFR9, IFNGR1, CREBBP, KBNA1, and PIAS1, as presented in Figure 7.

## 4. Discussion

We evaluated the functional and pathogenic sequences of missense SNPs of the human *STAT1* gene, utilizing 12 diverse in silico prediction tools (SIFT, PolyPhen2, PROVEAN, PANTHER, P MUT, PhD-SNP, SNPs&GO, SNAP2, and MutPred2). In silico prediction analysis identified six variants (I648T, V642D, R602W, L600P, I578N, and W504C) considered pathogenic and deleterious. These mutations have a major impact on the protein’s physicochemical characteristics, such as its size and charge hydrophobicity, which ultimately affect the protein’s stability and function and may have an impact on disease. Furthermore, we assessed the effect of missense SNPs on the stability of the STAT1 structure utilizing three stability prediction algorithms: I-Mutant3, MUpro, and DDMUT. All the variants revealed a reduction in stability by the three stability prediction tools (I-Mutant3, MUpro, and DDMUT). In general, we assumed that all missense SNPs in the *STAT1* gene were highly unstable in their protein structures, so they were selected for further structural bioinformatics analysis utilizing various tools to explore the consequences of tentatively destructive missense SNPs on STAT1 protein function. To evaluate the conservation profile, we used the ConSurf algorithm to represent the structural and functional conservation levels of all the amino acid residues of STAT1 protein. The ConSurf analysis revealed that the variant in position 602 is a functional residue in a highly conserved and exposed position. Structural residues in highly conserved and buried positions were identified in positions 600 and 578. The identified variants were found in a highly conserved region; this finding suggests that they might be involved in modifications of molecular mechanisms such as bond gain or loss.

*STAT1* GOF mutations with CMC were first described in 2001 and 2011, respectively; later, studies confirmed that *STAT1*-GOF mutations cause immunodeficiency and immune dysregulation, with a wide clinical spectrum [54].

Among the six SNPs identified linked to *STAT1* gene mutations in this study, some of these SNPs have been associated with diseases in previous studies, while others were projected to be so in this study using various computational tools. Population genetics and clinical studies are crucial to verifying the results of such research, even though utilizing computational techniques to analyze the impact of the SNPs may aid in identifying disease-related SNPs.

One mutation, namely L600P, has already been previously reported as a mutation in the *STAT1* gene in an infant who died of a viral-like illness associated with complete STAT1 deficiency and carried a homozygous nucleotide substitution (T→C) in exon 20, resulting in the substitution of a proline for a leucine at amino acid position 600 [55]. This mutation was found to be pathogenic using all the bioinformatics tools. I648T, V642D, R602W, I578N, and W504C were not reported previously.

Three mutations, namely L706S (rsRCV000009610), Q463H (VAR_065817), and E320Q (VAR_065816), have been reported as mutations in the *STAT1* gene. The two previously reported types of autosomal-dominant (AD) Mendelian susceptibility to mycobacterial disease (AD-MSMD) causing *STAT1* mutations are located in the tail segment domain (p.L706S) or in the DNA-binding domain (p.E320Q and p.Q463H) [56]. These mutations were not available in the dbSNP database. Two other SNPs (K637E) and (K673R) affecting the SH2 domain, which has been previously reported in two cases with AD-STAT1 deficiency in two unrelated patients from Japan and Saudi Arabia, were also not available in the dbSNPs database at the time of the analysis [56].

Two mutations linked to chronic mucocutaneous candidiasis are (T437I) and (Q271P). Q271P occurred within a specific pocket of the STAT1 coiled-coil domain, near residues essential for dephosphorylation, and was identified in a German patient who presented at 1 year of age with autosomal dominant chronic mucocutaneous candidiasis, showed signs of thyroid autoimmunity, and died at age 41 from squamous cell carcinoma [57,58]. These mutations were not available in the dbSNPs database.

The A267V variant in STAT1 has been reported in >10 individuals with chronic mucocutaneous candidiasis (CMC) and segregated with disease in 16 individuals from nine families [59]. This mutation was not present in the dbSNP database.

Interestingly, nsSNPs in the *STAT1* gene will ultimately affect and may disturb the normal function of other interacting genes. As our study was in detail, it provides all the information and analysis needed for the identification of the most damaging nsSNPs. Like ours, there are certain limitations in every study. Utilizing in silico technologies is now a crucial method for identifying disease-related SNPs. In this study, the *STAT1* gene underwent a thorough analysis utilizing 18 genetics analysis tools (10 computational tools and 8 AI-based methods) to determine the impact of nsSNPs on the protein’s structure and function.

Our study is based on computer tools and web servers, which are based on mathematical and statistical algorithms. Therefore, to confirm these results, experimental investigation is necessary.

## 5. Conclusions

Our study provides an insight about nsSNPs of the *STAT1* gene, its protein 3D structure, and its interactions with other genes, which might be helpful in future studies of *STAT1* in order to better understand its role in immunity and all related diseases.

## Figures and Tables

**Table 1:** List of nsSNPs that were predicted to have deleterious effect by SIFT, PolyPhen-2, Provean, and SNAP.

	SNP ID	Amino Acid Change	SIFT		Poly-Phen-2		Provean		SNAP2	
			Prediction	TI	Effect	Score	Effect	Score	Prediction	Score
1	rs1173266737	P728A	Deleterious	0	PB	0.974	Deleterious	−3.14	E	15
2	rs1374373369	D674V	Deleterious	0.01	PB	0.989	Deleterious	−7.327	E	47
3	rs771679419	Y668F	Deleterious	0	PS	0.609	Deleterious	−3.583	E	67
4	rs759271255	I648T	Deleterious	0.01	PB	1	Deleterious	−4.197	E	56
5	rs752542806	V642D	Deleterious	0	PB	0.994	Deleterious	−5.149	E	60
6	rs1387961263	L639F	Deleterious	0	PB	1	Deleterious	−3.489	E	7
7	rs1209841496	R602W	Deleterious	0	PB	1	Deleterious	−7.4	E	87
8	rs137852678	L600P	Deleterious	0	PB	1	Deleterious	−6.472	E	91
9	rs1398307167	P596Q	Deleterious	0.01	PS	0.951	Deleterious	−3.974	E	57
10	rs1398307167	P596L	Deleterious	0.01	PB	0.966	Deleterious	−5.853	E	58
11	rs767475430	I578N	Deleterious	0	PB	0.1	Deleterious	−6.392	E	80
12	rs113988352	I561T	Deleterious	0	PB	0.981	Deleterious	−4.143	E	38
13	rs1803838	P538L	Deleterious	0.03	PS	0.588	Deleterious	−3.097	E	10
14	rs916580554	W504C	Deleterious	0	PB	0.999	Deleterious	−11,937	E	48
15	rs1185249247	S503N	Deleterious	0	PS	0.949	Deleterious	−2.621	E	50
16	rs866554932	P481R	Deleterious	0.04	PB	0.1	Deleterious	−6.451	E	6
17	rs935654762	V455A	Deleterious	0	PB	0.997	Deleterious	−3.255	E	35
18	rs527393923	T450M	Deleterious	0	PB	0.1	Deleterious	−4.499	E	56
19	rs760409880	L448F	Deleterious	0	PS	0.816	Deleterious	−3.135	E	33
20	rs776192196	P326L	Deleterious	0	PB	0.996	Deleterious	−6.947	E	29
21	rs763976174	R304H	Deleterious	0.02	PS	0.850	Deleterious	−2.809	E	52
22	rs751403509	R304C	Deleterious	0	PB	0.1	Deleterious	−4.767	E	39
23	rs779371351	I248N	Deleterious	0	PB	0.1	Deleterious	−5.877	E	38
24	rs779371351	I248T	Deleterious	0	PB	0.1	Deleterious	−4.218	E	42
25	rs1017740241	C247Y	Deleterious	0	PB	0.1	Deleterious	−9.192	E	49
26	rs763588438	V149G	Deleterious	0.01	PB	0.987	Deleterious	−4.942	E	48
27	rs1482374494	A119T	Deleterious	0	PB	0.1	Deleterious	−3.113	E	26
28	rs756147217	P98S	Deleterious	0	PB	0.1	Deleterious	−5.885	E	48
29	rs865962653	S51L	Deleterious	0.01	PS	0.883	Deleterious	−4.4	E	34
30	rs781389511	A46T	Deleterious	0	PB	0.1	Deleterious	−2.543	E	1
31	rs34255470	I30T	Deleterious	0.02	PB	0.1	Deleterious	−3.391	E	17
32	rs11549696	P27T	Deleterious	0	PB	0.1	Deleterious	−6.503	E	64
33	rs1233778383	W4C	Deleterious	0	PB	1	Deleterious	−10.563	E	23

PB: probably damaging, PS: possibly damaging, E: effect.

**Table 2:** List of pathological nsSNPs predicted by PhD-SNP, SNPs and GO, P Mut, and PANTHER.

	nsSNP	Amino Acid Change	P MUT		PhD-SNP		SNP&GO		PANTHER	
			Prediction	Score	Prediction	RI	Prediction	RI	Effect	Preservation Time
1	rs1374373369	D674V	Disease	90%	Disease	9	Disease	8	probably damaging	1036
2	rs759271255	I648T	Disease	92%	Disease	6	Disease	6	probably damaging	842
3	rs752542806	V642D	Disease	87%	Disease	5	Disease	8	probably damaging	455
4	rs1209841496	R602W	Disease	93%	Disease	8	Disease	3	probably damaging	1237
5	rs137852678	L600P	Disease	93%	Disease	9	Disease	8	probably damaging	1036
6	rs767475430	I578N	Disease	93%	Disease	8	Disease	6	probably damaging	1237
7	rs916580554	W504C	Disease	91%	Disease	6	Disease	8	probably damaging	750
8	rs527393923	T450M	Disease	86%	Disease	1	Disease	2	probably damaging	1036
9	rs865962653	S51L	Disease	88%	Disease	5	Disease	1	probably damaging	750

**Table 3:** MutPred probability values of deleterious and pathogenic nsSNPs identified in STAT1.

	SNP ID	Amino Acid Change	MutPred 2 Score	Affected PROSITE and ELM Motifs	Molecular Mechanisms	Probability	*p*-Value
1	rs1374373369	D674V	0.813		Gain of Strand Gain of Acetylation at K673	0.26 0.25	0.04 0.01
2	rs759271255	I648T	0.893		Altered Stability	0.16	0.02
3	rs752542806	V642D	0.867	ELME000063 ELME000085 ELME000147 ELME000155 ELME000220 ELME000233	Altered Ordered interface	0.35	4.2 × 10^−3^
					Gain of Relative solvent Accessibility	0.30	7.3 × 10^−3^
					Altered Transmembrane protein	0.18	8.6 × 10^−3^
					Altered DNA binding	0.15	0.04
4	rs1209841496	R602W	0.896	- ELME000328 ELME000052 ELME000062	Gain of Strand	0.27	0.02
					Altered Stability	0.09	0.05
5	rs137852678	L600P	0.965	ELME000052 ELME000328	Gain of Intrinsic disorder	0.31	0.04
					Altered Stability	0.28	6.6 × 10^−3^
6	rs767475430	I578N	0.936	PS00008			
7	rs916580554	W504C	0.807	ELME000197			
8	rs527393923	T450M	0.373				
9	rs865962653	S51L	0.665	ELME000063 ELME000147 ELME000336	Altered transmembrane protein	0.23	2.4 × 10^−3^

*p*-values ≤ 0.05.

**Table 4:** Deleterious and pathogenic nsSNPs predicted to have a significant decrease on protein stability by I-MUTANT 3.0 algorithm, MUpro, and DDMUT.

	SNP ID	Amino Acid Change	I Mutant 3			MUpro		DDMUT	
			Stability	RI	DDG (kcal/mol)	Stability	DDG (kcal/mol)	Stability	DDG (kcal/mol)
1	rs759271255	I648T	Decrease	9	−2.43	Decrease	−2.4802937	Destabilizing	−2.93
2	rs752542806	V642D	Decrease	8	−1.85	Decrease	−1.8071037	Destabilizing	−1.11
3	rs1209841496	R602W	Decrease	3	−0.20	Decrease	−1.0486884	Destabilizing	−0.19
4	rs137852678	L600P	Decrease	3	−1.54	Decrease	−1.6074419	Destabilizing	−3.06
5	rs767475430	I578N	Decrease	5	−1.92	Decrease	−0.98144877	Destabilizing	−0.84
6	rs916580554	W504C	Decrease	8	−1.41	Decrease	−0.86533645	Destabilizing	−0.73

**Table 5:** Alpha-Missense prediction of the pathogenic nsSNPs in STAT1.

	SNP ID	Substitution	Alpha-Missense Pathogenicity	Alpha-Missense Prediction
1	rs759271255	I648T	0.9875	Likely Pathogenic
2	rs752542806	V642D	0.9916	Likely Pathogenic
3	rs1209841496	R602W	0.9982	Likely Pathogenic
4	rs137852678	L600P	0.9998	Likely Pathogenic
5	rs767475430	I578N	0.9986	Likely Pathogenic
6	rs916580554	W504C	0.9815	Likely Pathogenic

**Table 6:** Conservation profile of most damaging nsSNPs of STAT1.

	SNP ID	Amino Acid Change	Conservation Score	Prediction
1	rs759271255	I648T	8	Conserved and buried
2	rs752542806	V642D	6	Buried
3	rs120984149	R602W	9	(functional residues), highly conserved and exposed
4	rs137852678	L600P	9	(structural residues), highly conserved and buried
5	rs767475430	I578N	9	(structural residues), highly conserved and buried

**Table 7:** Changes in physical properties between wild-type and mutant residues predicted by project hope.

	SNPs	Difference in Size	Difference in Charge	Difference in Hydrophobicity	Disrupt Hydrogen Bond	Affect Contact with Ligand Molecules
1	I648T	Yes	No	Yes	No	Yes
2	V642D	Yes	No	Yes	No	Yes
3	R602W	Yes	No	Yes	No	Yes
4	L600P	Yes	No	Yes	No	No
5	I578N	Yes	No	Yes	Yes	Yes
6	W504C	Yes	No	Yes	No	Yes

**Table 8:** Domain regions of the selected most damaging nsSNPs in STAT1.

STAT1 Domains (Position)	SNPs
STAT1, SH2 domain (557–707)	Y668F, I648T, V642D, R602W, and L600P
STAT1 transcription factor, DNA binding domain (323–458)	R304C
SH2 domain (578–638)	I578N
Src homology 2 (SH2) domain profile (573–670)	I578N

## Data Availability

The original contributions presented in this study are included in the article/Supplementary Materials. Further inquiries can be directed to the corresponding author.

## Supplementary Materials

The following supporting information can be downloaded at: https://www.mdpi.com/article/10.3390/genes16030303/s1, Figure S1: Shows the percentages of the SNPs in *STAT1* gene; Figure S2: Heat map generated by alpha-missense, shows the variations in *STAT1* gene; Figure S3: Conservation profile of amino acids in STAT1protein; Figure S4: Protein 2D structure of human STAT1predicted by AlphaFold2; Figure S5: Effect of the six most deleterious nsSNPs on the STAT1 protein structure; Figure S6: Difference in ionic interactions between the wild-type (A) and mutant residues (B) in I648T; Table S1: List of nsSNPs that were predicted to have deleterious effect by SIFT, PolyPhen-2, Provean and SNAP2; Table S2: MutPred probability values of deleterious and pathogenic nsSNPs identified in STAT1; Table S3: Deleterious and pathogenic ns SNPs were predicted to have significant decrease on protein stability by I-MUTANT 3.0 algorithm, MUpro, and DDMUT; Table S4: Shows Alpha-missense prediction of the pathogenic nsSNPs in STAT1; Table S5: Conservation profile of most damaging nsSNPs of STAT1; Table S6: Changes in physical properties between wild-type and mutant residues predicted by project hope; Table S7: Domain regions of the selected most damaging nsSNPs in STAT1.
