# Supplementary material for: Integrating Artificial Intelligence and Bioinformatics Methods to Identify Disruptive STAT1 Variants Impacting Protein Stability and Function
Source: Genes (Basel). 2025 Mar 1;16(3):303. doi: 10.3390/genes16030303 (PMC11942549; doi:10.3390/genes16030303)
Supplement: Supplementary file 1 [file genes-16-00303-s001.zip › final supplementary tables STAT1 paper.docx]

|  | **SNP ID** | **Amino-acid change** | **SIFT** | | **Poly-Phen-2** | | **Provean** | | **SNAP2** | |
| --- | --- | --- | --- | --- | --- | --- | --- | --- | --- | --- |
|  |  |  | **Prediction** | **TI** | **Effect** | **Score** | **Effect** | **Score** | **Prediction** | **Score** |
| 1 | rs1173266737 | P728A | Deleterious | 0 | PB | 0.974 | Deleterious | -3.14 | E | 15 |
| 2 | [rs1374373369](https://www.ncbi.nlm.nih.gov/projects/SNP/snp_ref.cgi?rs=1374373369) | D674V | Deleterious | 0.01 | PB | 0.989 | Deleterious | -7.327 | E | 47 |
| 3 | rs771679419 | Y668F | Deleterious | 0 | PS | 0.609 | Deleterious | -3.583 | E | 67 |
| 4 | rs759271255 | I648T | Deleterious | 0.01 | PB | 1 | Deleterious | -4.197 | E | 56 |
| 5 | rs752542806 | V642D | Deleterious | 0 | PB | 0.994 | Deleterious | -5.149 | E | 60 |
| 6 | rs1387961263 | L639F | Deleterious | 0 | PB | 1 | Deleterious | -3.489 | E | 7 |
| 7 | rs1209841496 | R602W | Deleterious | 0 | PB | 1 | Deleterious | -7.4 | E | 87 |
| 8 | rs137852678 | L600P | Deleterious | 0 | PB | 1 | Deleterious | -6.472 | E | 91 |
| 9 | rs1398307167 | P596Q | Deleterious | 0.01 | PS | 0.951 | Deleterious | -3.974 | E | 57 |
| 10 | rs1398307167 | P596L | Deleterious | 0.01 | PB | 0.966 | Deleterious | -5.853 | E | 58 |
| 11 | rs767475430 | I578N | Deleterious | 0 | PB | 0.1 | Deleterious | -6.392 | E | 80 |
| 12 | rs113988352 | I561T | Deleterious | 0 | PB | 0.981 | Deleterious | -4.143 E 38 | | |
| 13 | rs1803838 | P538L | Deleterious | 0.03 | PS | 0.588 | Deleterious | -3.097 | E | 10 |
| 14 | rs916580554 | W504C | Deleterious | 0 | PB | 0.999 | Deleterious | -11,937 | E | 48 |
| 15 | rs1185249247 | S503N | Deleterious | 0 | PS | 0.949 | Deleterious | -2.621 | E | 50 |
| 16 | rs866554932 | P481R | Deleterious | 0.04 | PB | 0.1 | Deleterious | -6.451 | E | 6 |
| 17 | rs935654762 | V455A | Deleterious | 0 | PB | 0.997 | Deleterious | -3.255 | E | 35 |
| 18 | rs527393923 | T450M | Deleterious | 0 | PB | 0.1 | Deleterious | -4.499 | E | 56 |
| 19 | rs760409880 | L448F | Deleterious | 0 | PS | 0.816 | Deleterious | -3.135 | E | 33 |
| 20 | rs776192196 | P326L | Deleterious | 0 | PB | 0.996 | Deleterious | -6.947 | E | 29 |
| 21 | rs763976174 | R304H | Deleterious | 0.02 | PS | 0.850 | Deleterious | -2.809 | E | 52 |
| 22 | rs751403509 | R304C | Deleterious | 0 | PB | 0.1 | Deleterious | -4.767 | E | 39 |
| 23 | rs779371351 | I248N | Deleterious | 0 | PB | 0.1 | Deleterious | -5.877 | E | 38 |
| 24 | rs779371351 | I248T | Deleterious | 0 | PB | 0.1 | Deleterious | -4.218 | E | 42 |
| 25 | rs1017740241 | C247Y | Deleterious | 0 | PB | 0.1 | Deleterious | -9.192 | E | 49 |
| 26 | rs763588438 | V149G | Deleterious | 0.01 | PB | 0.987 | Deleterious | -4.942 | E | 48 |
| 27 | rs1482374494 | A119T | Deleterious | 0 | PB | 0.1 | Deleterious | -3.113 | E | 26 |
| 28 | rs756147217 | P98S | Deleterious | 0 | PB | 0.1 | Deleterious | -5.885 | E | 48 |
| 29 | rs865962653 | S51L | Deleterious | 0.01 | PS | 0.883 | Deleterious | -4.4 | E | 34 |
| 30 | rs781389511 | A46T | Deleterious | 0 | PB | 0.1 | Deleterious | -2.543 | E | 1 |
| 31 | rs34255470 | I30T | Deleterious | 0.02 | PB | 0.1 | Deleterious | -3.391 | E | 17 |
| 32 | rs11549696 | P27T | Deleterious | 0 | PB | 0.1 | Deleterious | -6.503 | E | 64 |
| 33 | rs1233778383 | W4C | Deleterious | 0 | PB | 1 | Deleterious | -10.563 | E | 23 |

Table S 1. List of nsSNPs that were predicted to have deleterious effect by SIFT, PolyPhen-2, Provean and SNAP2

Table S2. MutPred probability values of deleterious and pathogenic nsSNPs identified in STAT1

|  | **nsSNP ID** | **Amino acid**  **change** | **MutPred 2 score** | **Affected PROSITE and ELM Motifs** | **Molecular mechanisms P-values <= 0.05** | **Probability** | **P-value** |
| --- | --- | --- | --- | --- | --- | --- | --- |
| 1 | rs1374373369 | D674V | 0.813 | - | Gain of Strand | 0 .26 | 0.04 |
|  |  |  |  |  | Gain of Acetylation at K673 | 0.25 | 0.01 |
| 2 | rs759271255 I648T | | 0.893 |  | Altered Stability | 0.16 | 0.02 |
| 3 | rs752542806 V642D | | 0.867. | ELME000063 ELME000085  ELME000147  ELME000155  ELME000220  ELME000233 | Altered Ordered interface | 0.35 | 4.2e-03 |
|  |  |  |  |  | Gain of Relative solvent  Accessibility | 0.30 | 7.3e-03 |
|  |  |  |  |  | Altered Transmembrane protein | 0.18 | 8.6e-03 |
|  |  |  |  |  | Altered DNA binding | 0.15 | 0.04 |
| 4 | rs1209841496 | R602W | 0.896 | -  ELME000328  ELME000052  ELME000062 | Gain of Strand | 0.27 | 0.02 |
|  |  |  |  |  | Altered Stability | 0.09 | 0.05 |
| 5 | rs137852678 | L600P | 0.965 | ELME000052  ELME000328 | Gain of Intrinsic disorder | 0.31 | 0.04 |
|  |  |  |  |  | Altered Stability | 0.28 | 6.6e-03 |
| 6 | rs767475430 | I578N 0.936 | | PS00008 | - | - | - |
| 7 | rs916580554 | W504C 0.807 | | ELME000197 | - | - | - |
| 8 | rs527393923 | T450M 0.373 | | - | - | - | - |
| 9 | rs865962653 | S51L | 0.665 | ELME000063ELME000147  ELME000336 | Altered transmembrane protein | 0.23 | 2.4e-03 |

Table S3. Deleterious and pathogenic ns SNPs were predicted to have significant decrease on protein stability by I-MUTANT 3.0 algorithm, MUpro, and DDMUT

|  | **SNP ID** | **Amino acid**  **change** | **I mutant 3** | | | **MUpro DDMUT** | |
| --- | --- | --- | --- | --- | --- | --- | --- |
|  |  |  | **Stability** | **RI** | **DDG**  **(kcal/mol)** | **Stability** | **DDG Stability DDG**  **(kcal/mol) (kcal/mol)** |
| 1 | rs759271255 | I648T | Decrease | 9 | -2.43 | Decrease | -2.4802937 Destabilizing -2.93 |
| 2 | rs752542806 | V642D | Decrease | 8 | -1.85 | Decrease | -1.8071037 Destabilizing -1.11 |
| 3 | rs1209841496 | R602W | Decrease | 3 | -0.20 | Decrease | -1.0486884 Destabilizing: -0.19 |
| 4 | rs137852678 | L600P | Decrease | 3 | -1.54 | Decrease | -1.6074419 Destabilizing -3.06 |
| 5 | rs767475430 | I578N | Decrease | 5 | -1.92 | Decrease | -0.98144877 Destabilising -0.84 |
| 6 | rs916580554 | W504C | Decrease | 8 | -1.41 | Decrease | -0.86533645 Destabilizing -0.73 |

Table S4. Shows Alpha-missense prediction of the pathogenic nsSNPs in STAT1

|  | **SNP ID** | **Substitution** | **Alpha-missense pathogenicity** | **Alpha-missense prediction** |
| --- | --- | --- | --- | --- |
| 1 | rs759271255 | I648T | 0.9875 | Likely Pathogenic |
| 2 | rs752542806 | V642D | 0.9916 | Likely Pathogenic |
| 3 | rs1209841496 | R602W | 0.9982 | Likely Pathogenic |
| 4 | rs137852678 | L600P | 0.9998 | Likely Pathogenic |
| 5 | rs767475430 | I578N | 0.9986 | Likely Pathogenic |
| 6 | rs916580554 | W504C | 0.9815 | Likely Pathogenic |
|  |  |  |  |  |

Table S5. Conservation profile of most damaging nsSNPs of STAT1

| **No** | **SNP ID** | **Amino acid**  **change** | **Conservation**  **score** | **Prediction** |
| --- | --- | --- | --- | --- |
| 1 | rs759271255 | I648T | 8 | Conserved and buried |
| 2 | rs752542806 | V642D | 6 | Buried |
| 3 | rs120984149 | R602W | 9 | (functional residues), highly conserved and exposed |
| 4 | rs137852678 | L600P | 9 | (structural residues), highly conserved and buried |
| 5 | rs767475430 | I578N | 9 | (structural residues), highly conserved and buried |
| 6 | rs916580554 | W504C | 8 | Conserved and buried |
|  |  |  |  |  |

Table S6. Changes in physical properties between wild-type and mutant residues predicted by project hope

|  | **SNPs** | **Differ in size** | **Difference in charge** | **Difference in hydrophobicity** | **Disrupt hydrogen bond** | **Affect contact with ligand molecules** |  |
| --- | --- | --- | --- | --- | --- | --- | --- |
|  |  |  |  |  |  |  |  |
| 1 | I648T | Yes | No | Yes | No | Yes |  |
| 2 | V642D | Yes | No | Yes | No | Yes |  |
| 3 | R602W | Yes | No | Yes | No | Yes |  |
| 4 | L600P | Yes | No | Yes | No | No |  |
| 5 | I578N | Yes | No | Yes | Yes | Yes |  |
| 6 | W504C | Yes | No | Yes | No | Yes |  |

Table S7. Domain regions of the selected most damaging nsSNPs in STAT1

| STAT1 domains (position) | SNPs |
| --- | --- |
| STAT1, SH2 domain (557–707) | Y668F, I648T , V642D, R602W, and L600P |
| STAT1 transcription factor, DNA binding domain (323–458) | R304C |
| SH2 domain (578–638) | I578N |
| Src homology 2 (SH2) domain profile (573-670) | I578N |
