# Supplementary material for: Integrating Artificial Intelligence and Bioinformatics Methods to Identify Disruptive STAT1 Variants Impacting Protein Stability and Function
Source: Genes (Basel). 2025 Mar 1;16(3):303. doi: 10.3390/genes16030303 (PMC11942549; doi:10.3390/genes16030303)
Supplement: Supplementary file 1 [file genes-16-00303-s001.zip › final supplementary_figures STAT1 paper.docx]

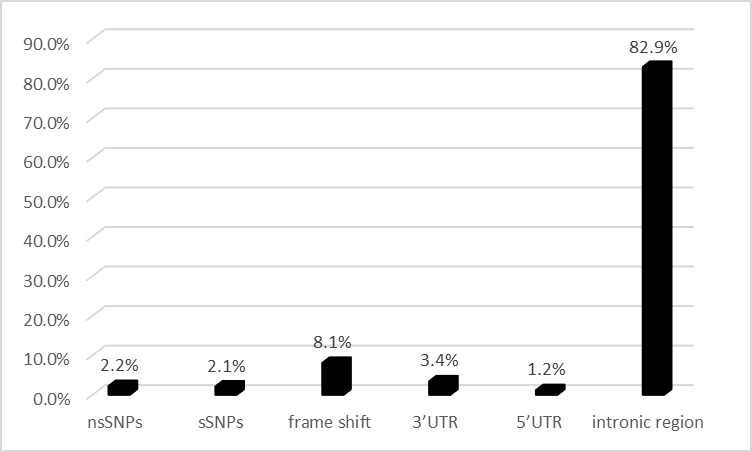


Figure S1. Shows the percentages of the SNPs in *STAT1* gene (nsSNPs: 1.8%; 3’UTR SNPs: 3.4%; 5’UTR SNPs: 1.2%; Other SNPs: 93.6%)


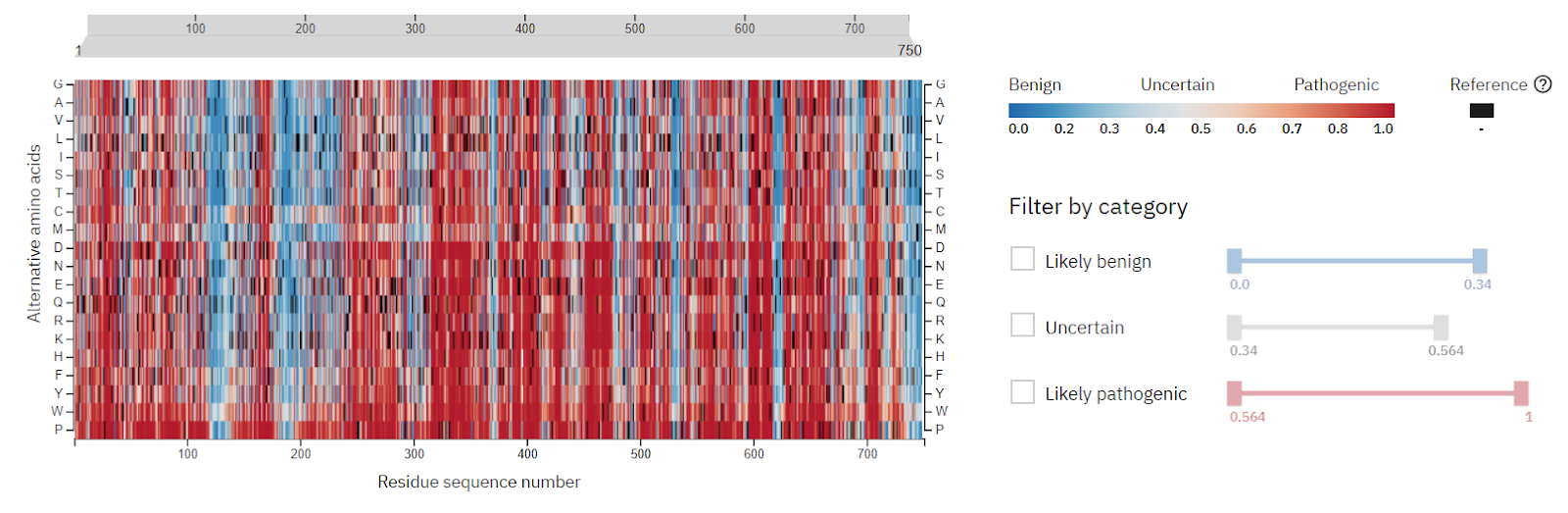


Figure S2. Heat map generated by alpha-missense, shows the variations in *STAT1* gene.


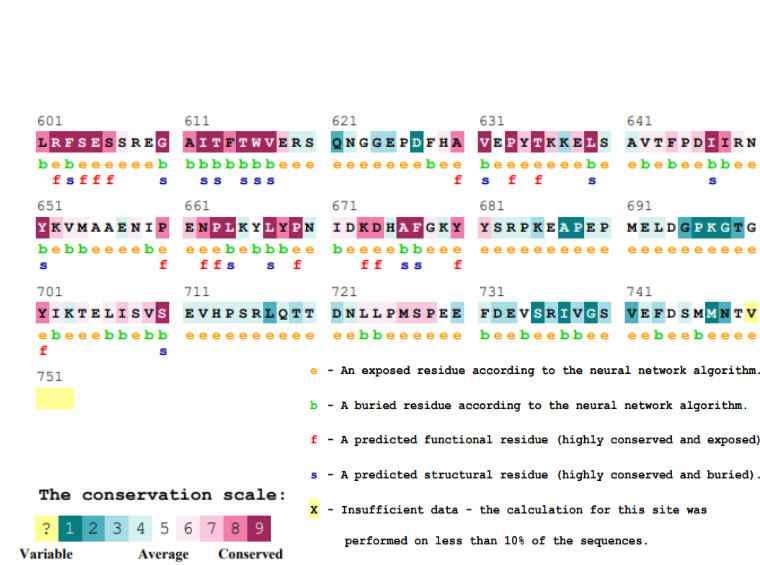


Figure S3. Conservation profile of amino acids in STAT1protein


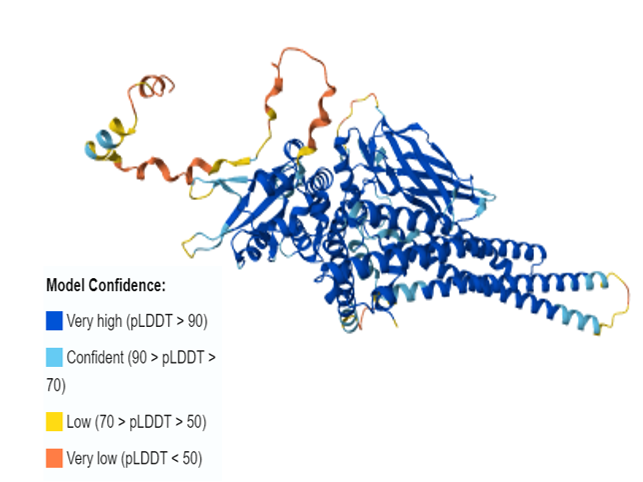


Figure S4. Protein 2D structure of human STAT1predicted by AlphaFold2


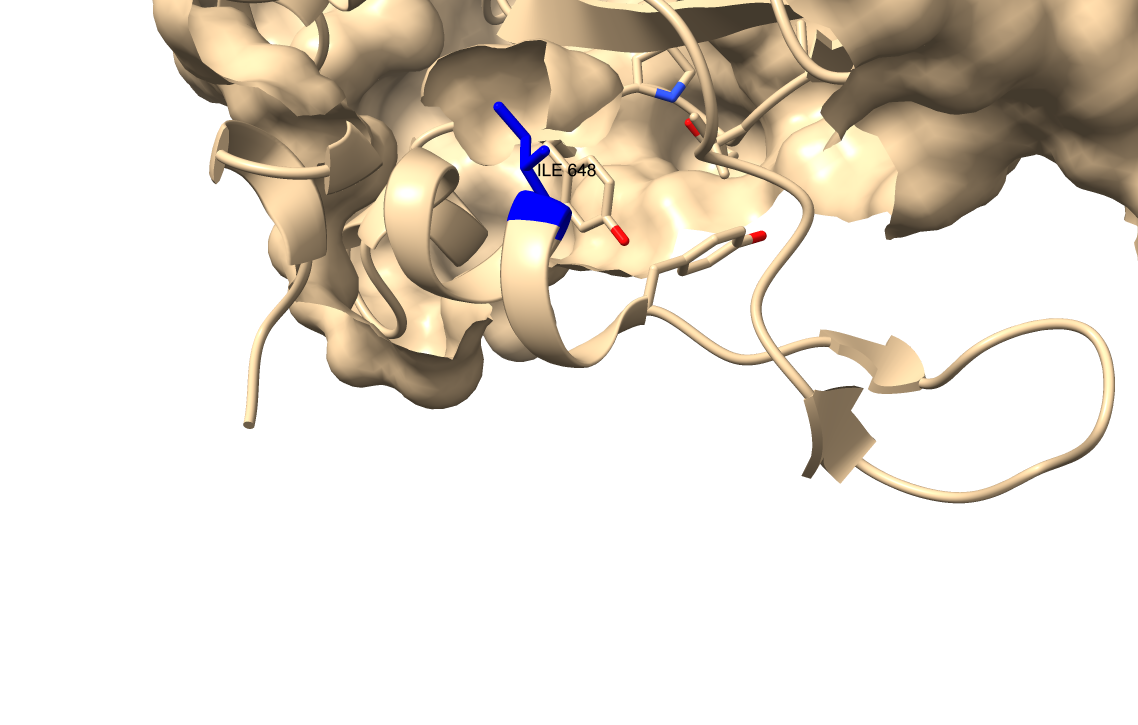


Wild type amino acid at position 648.


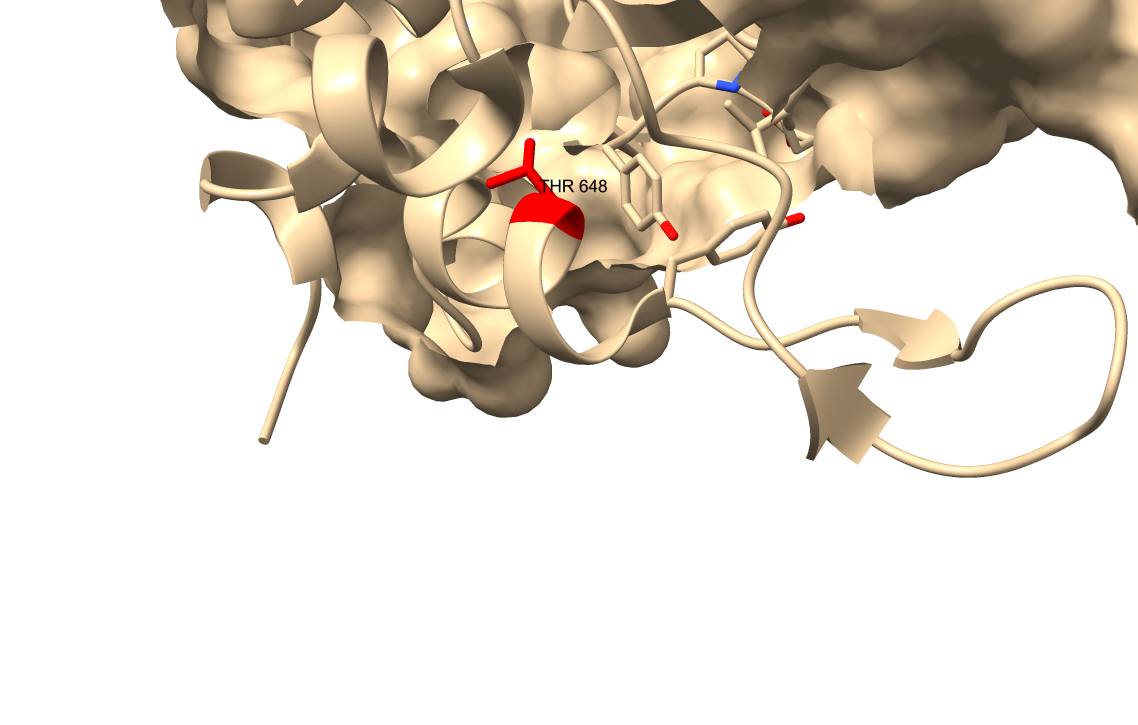


Mutant residue is at position 648.


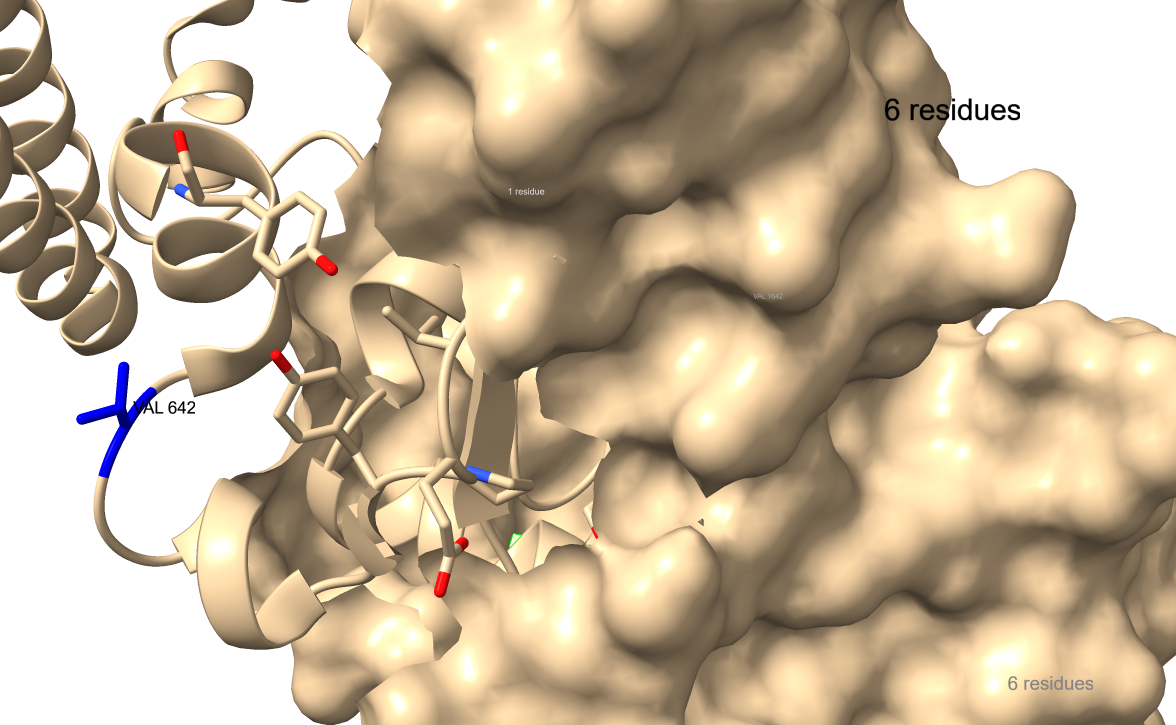


Wild-type amino acid at position 642.


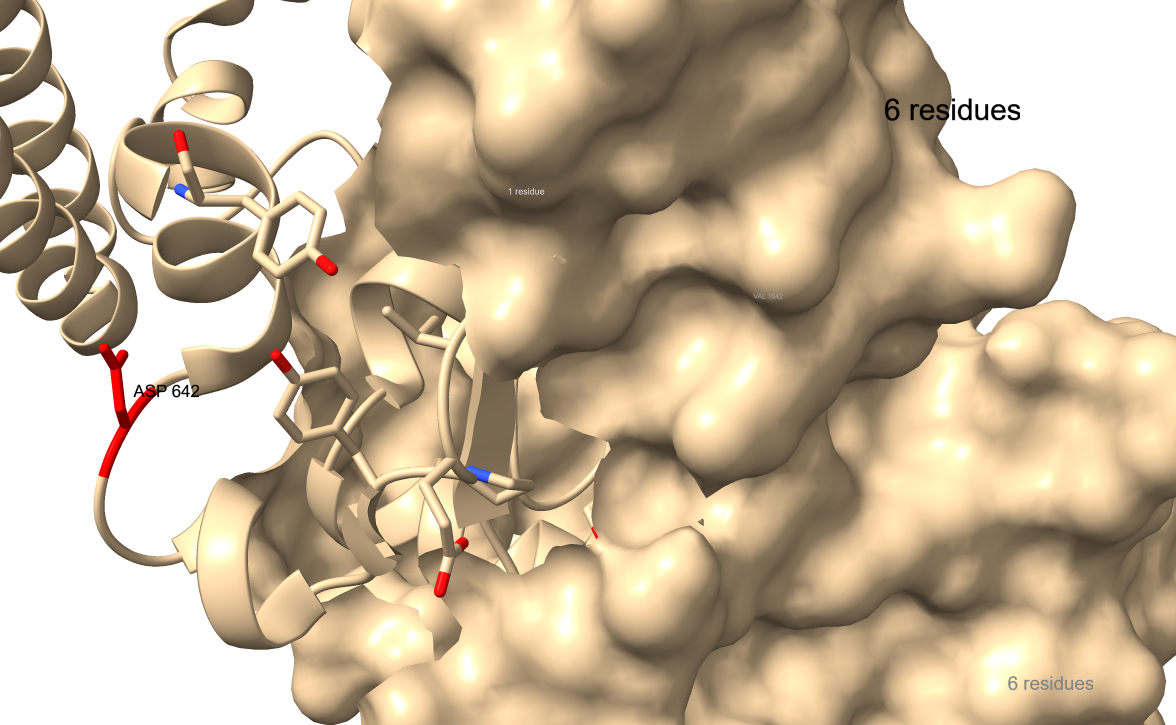


Mutant residue is at position 642.


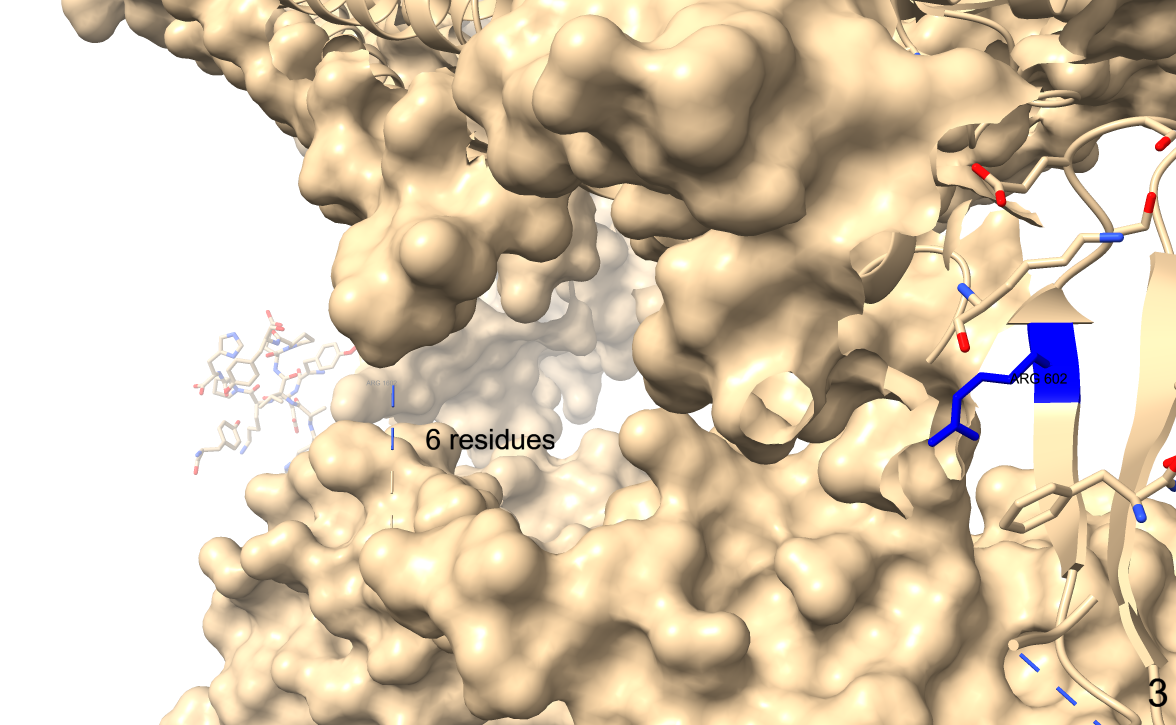


Wild type amino acid at position 602.


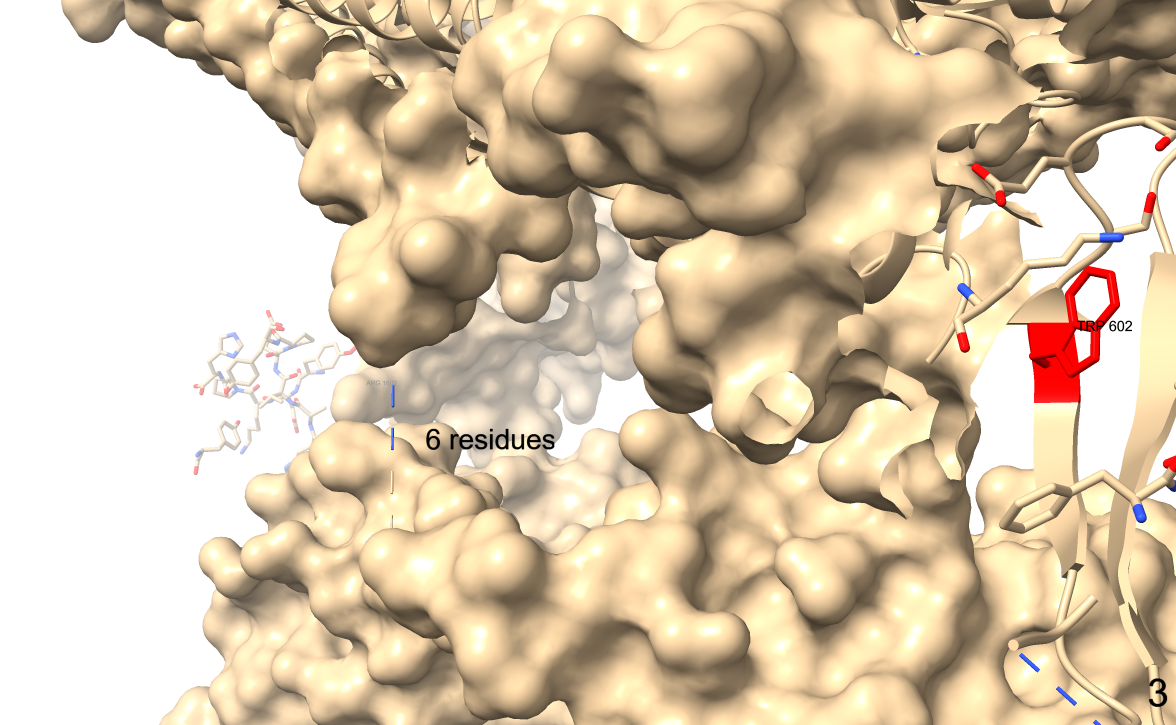


Mutant residue at position 602.


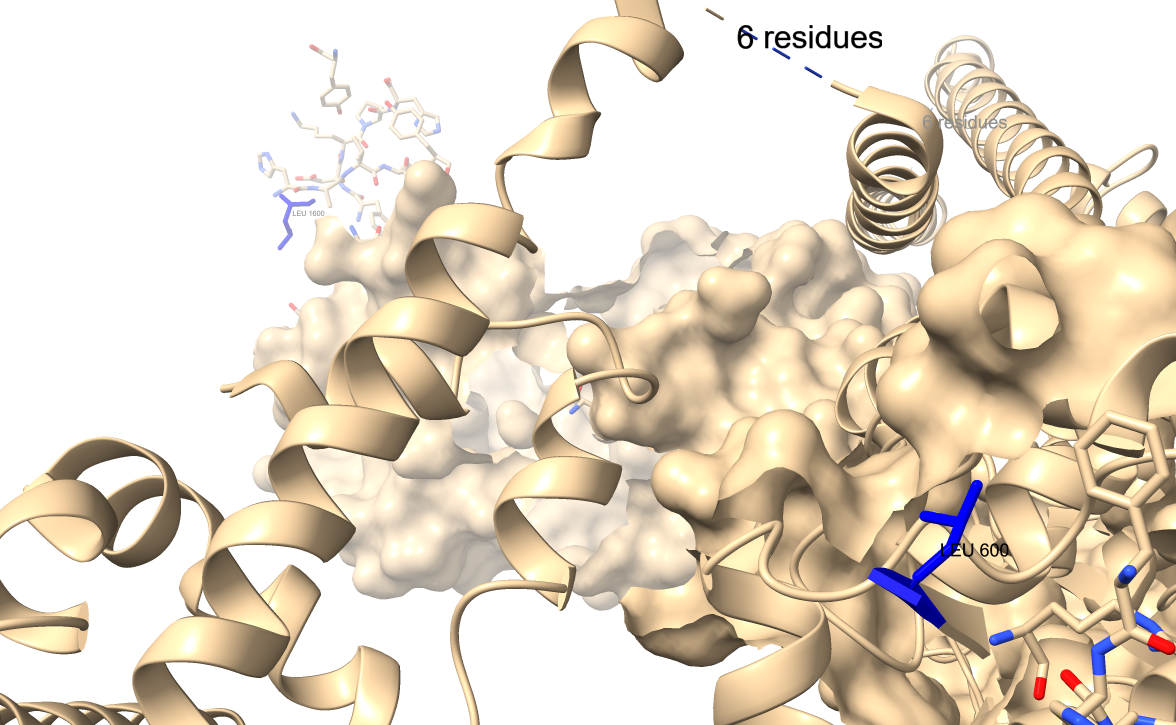


Wild type amino acid at position 600.


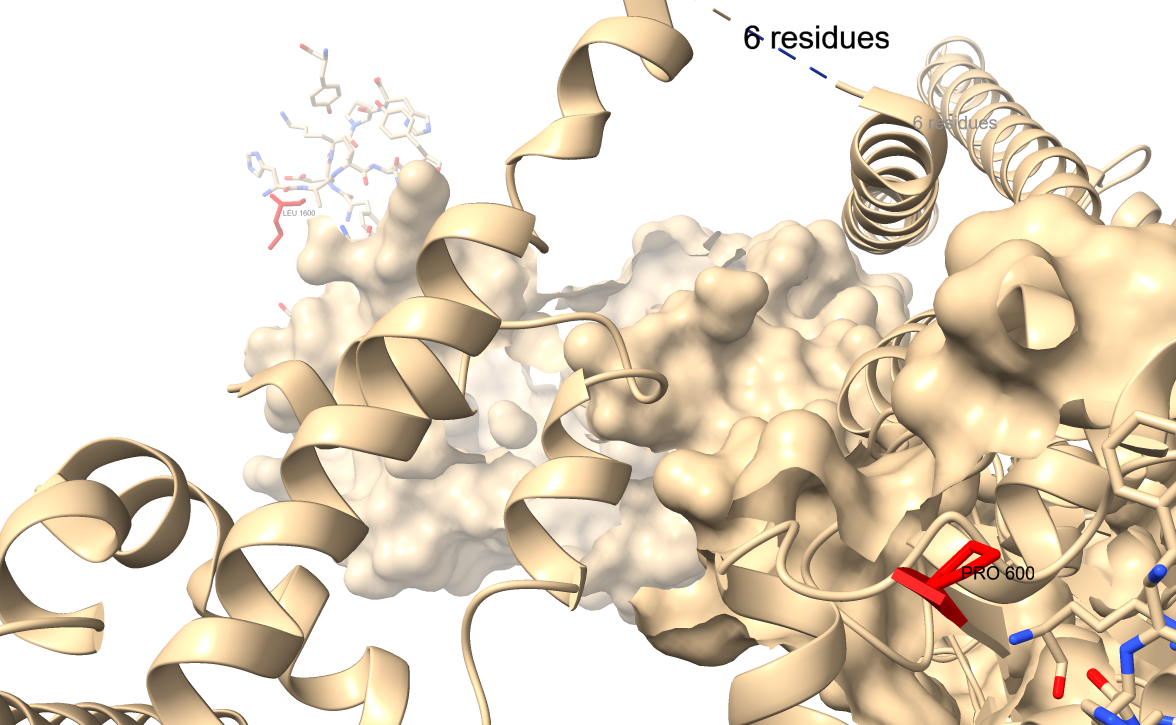


Mutant residue at position 600.


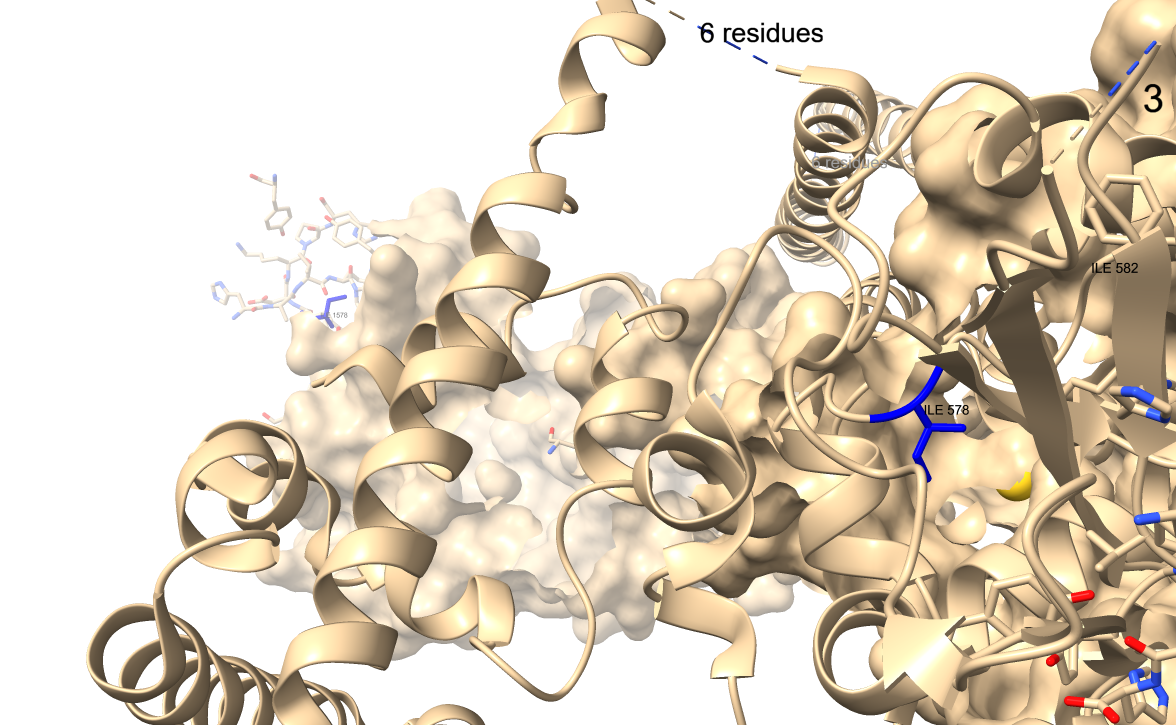


Wild-type amino acid at position 578.


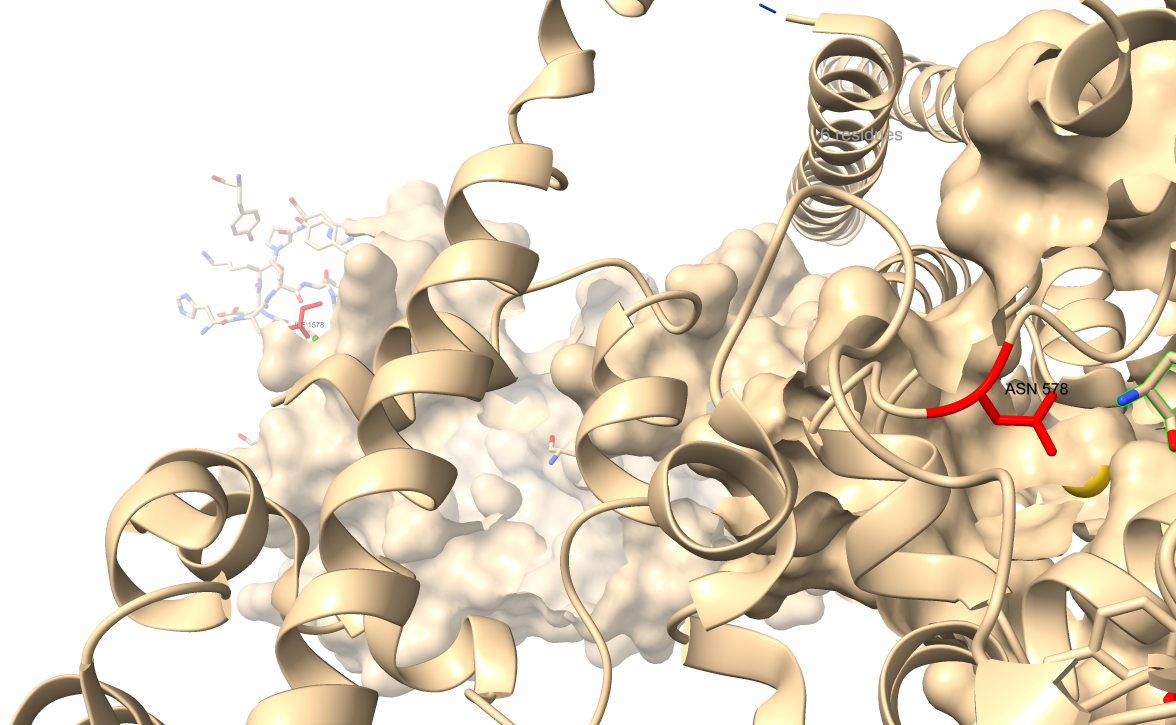


Mutant residue at position 578.


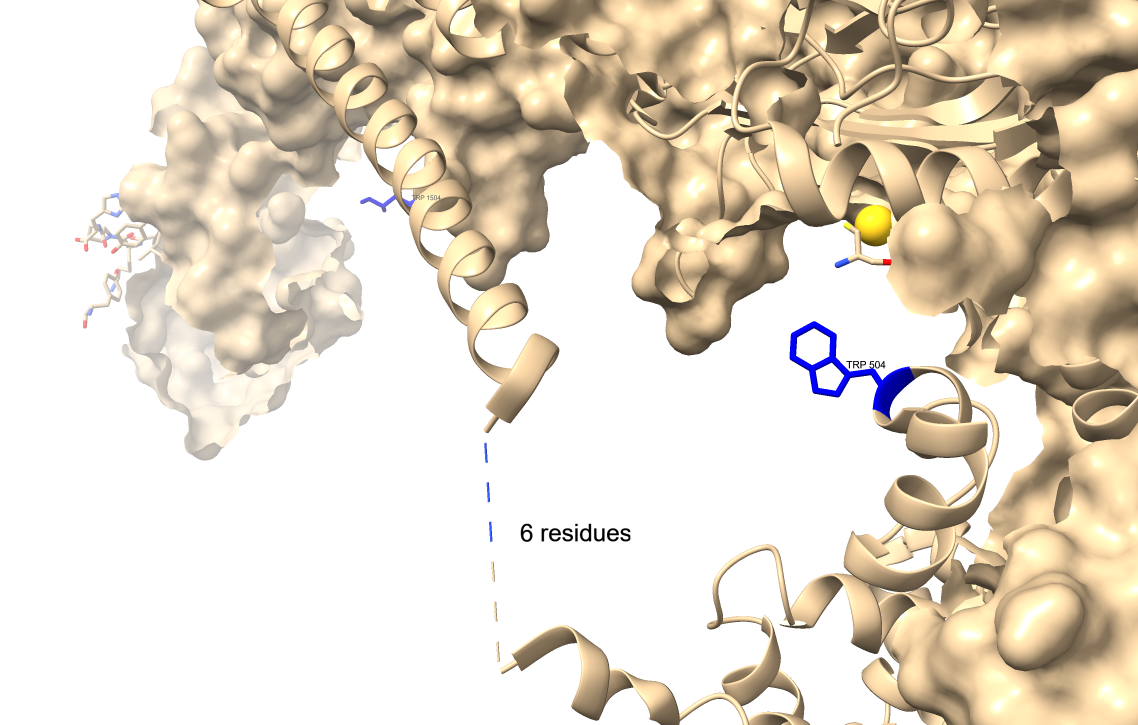


Wild type amino acid at position 504.


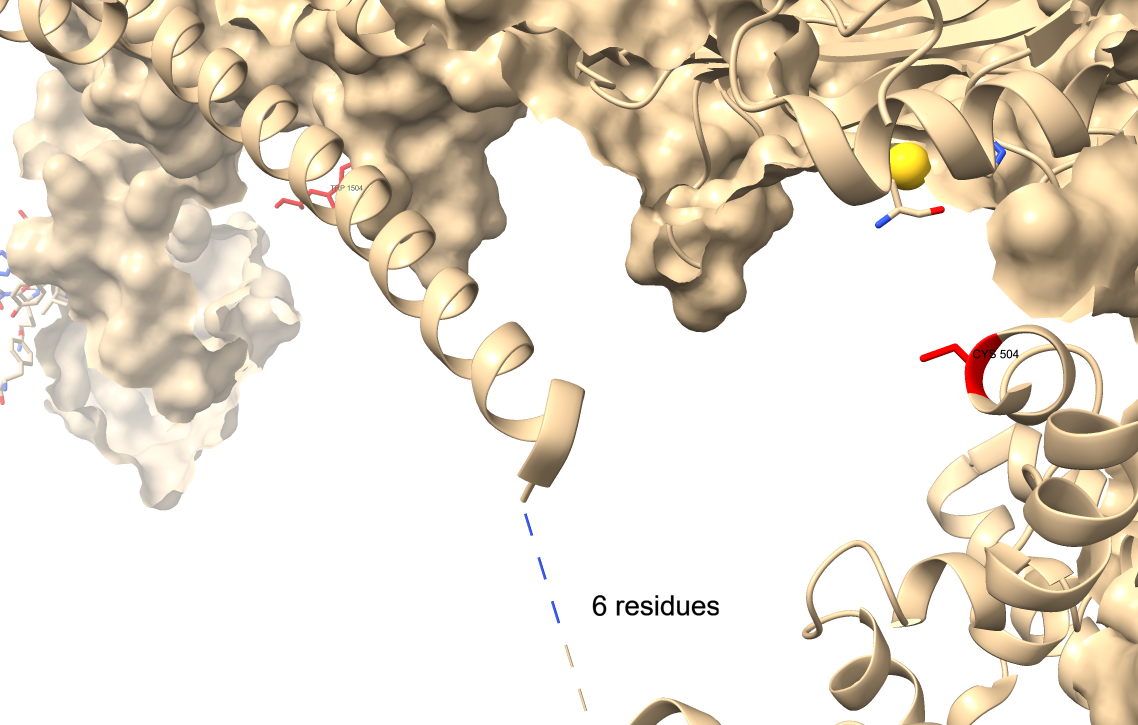


Mutant residue at position 504.

Figure S5. Effect of the six most deleterious nsSNPs on the STAT1 protein structure. ChimeraX software was used to visualize the 3D structure.


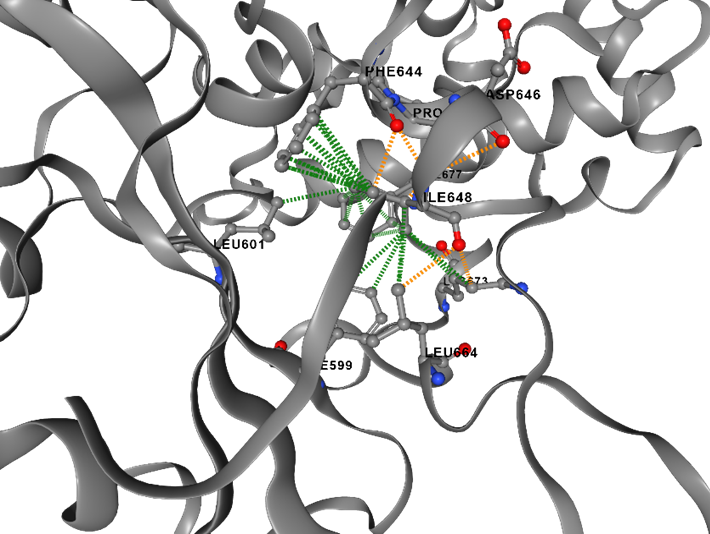

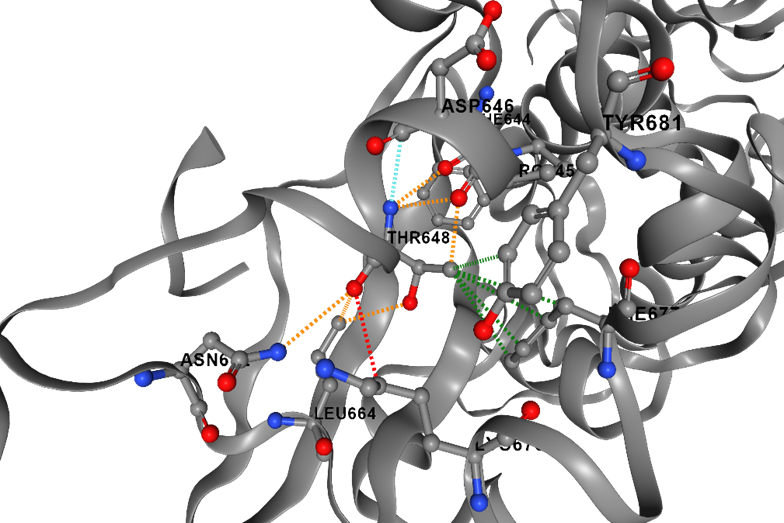


(A)I648T - wild type (B) I648T, mutant type


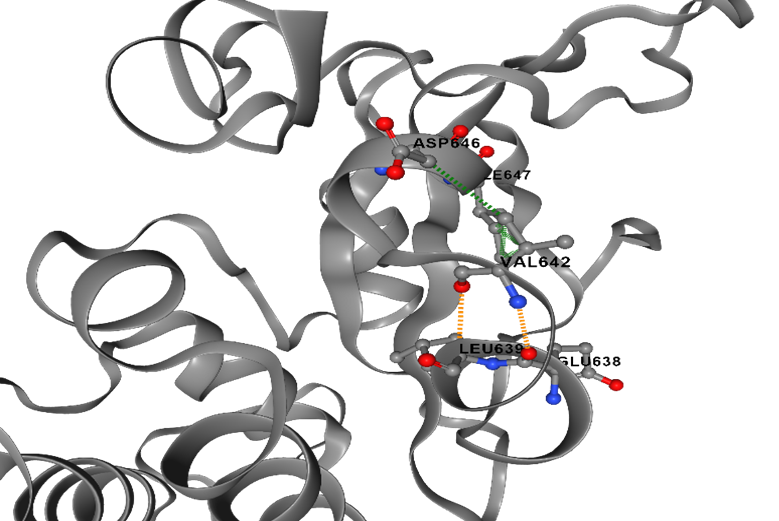

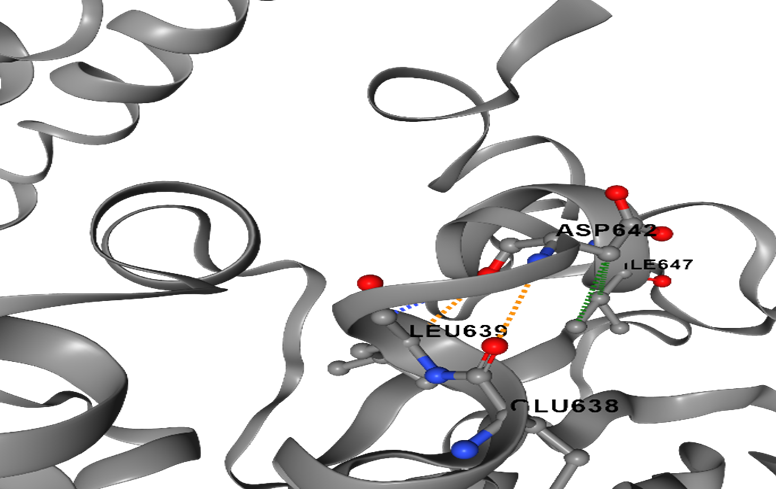


(A)V642D - wild type (B) V642D, mutant residue


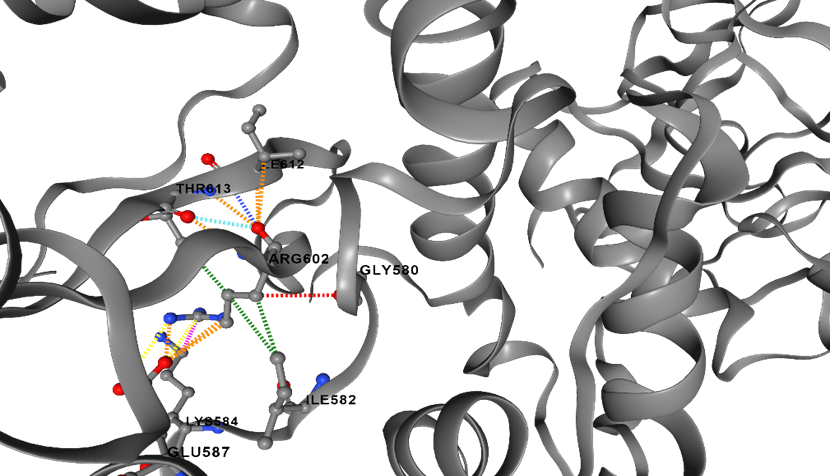

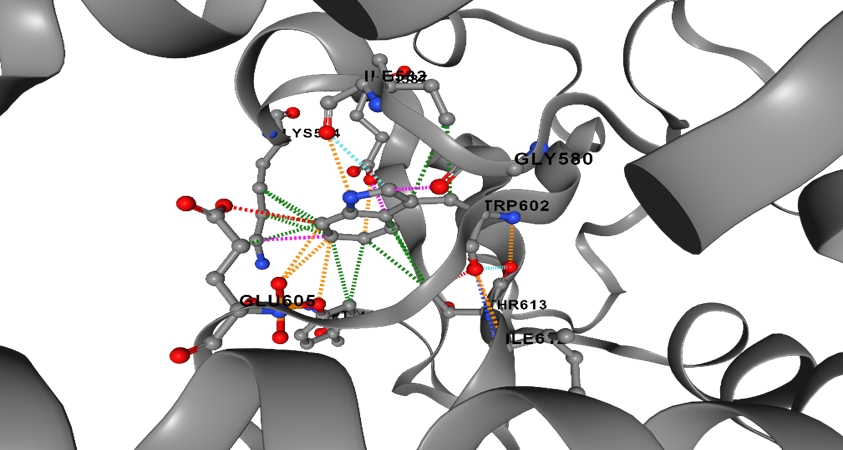


(A)R602W - wild type (B) R602W, mutant residue


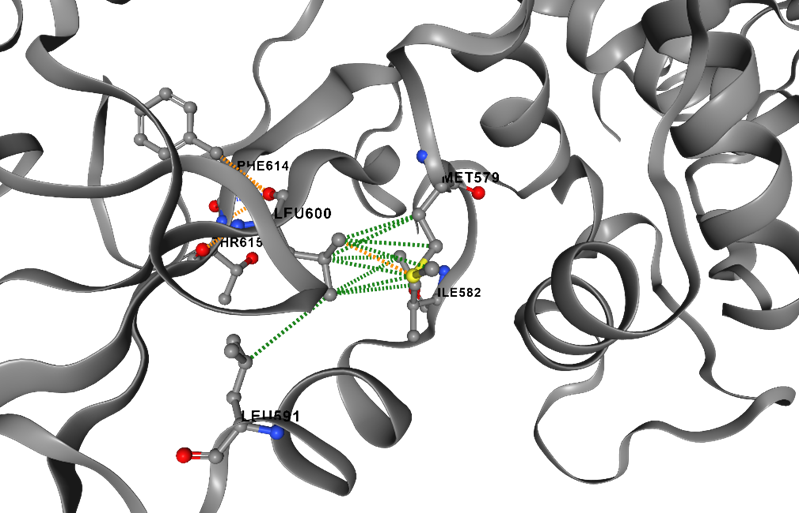

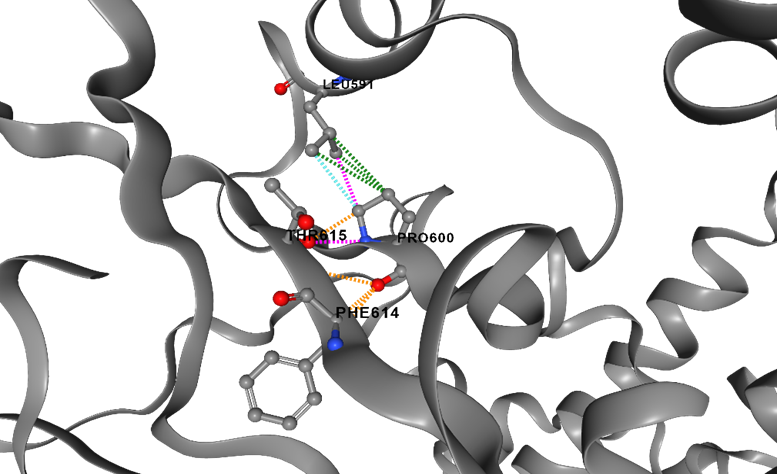


(A)L600P - wild type (B) L600P mutant type


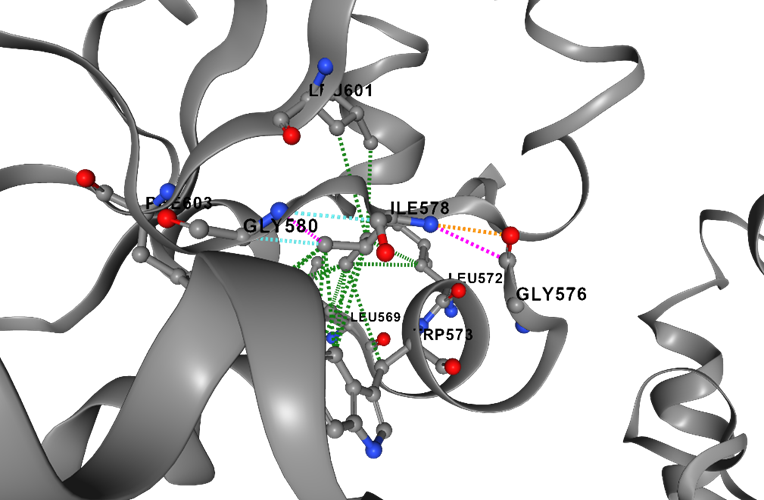

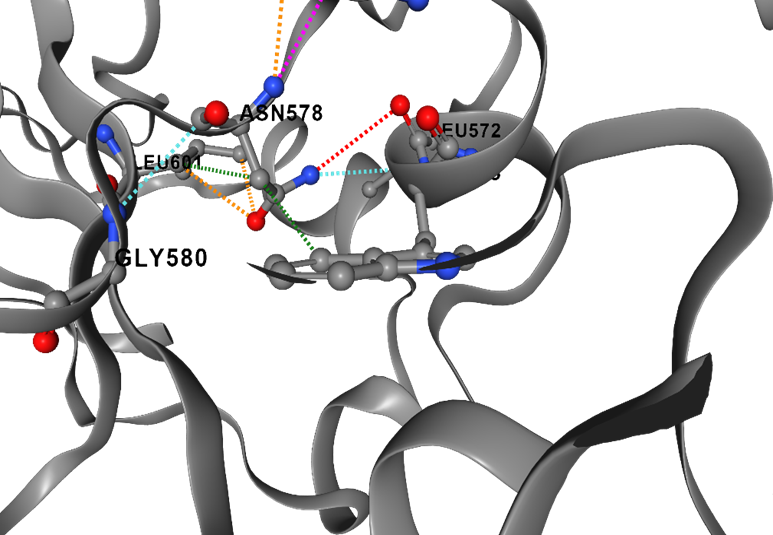


(A)I578N - wild type (B) I578N, mutant residue


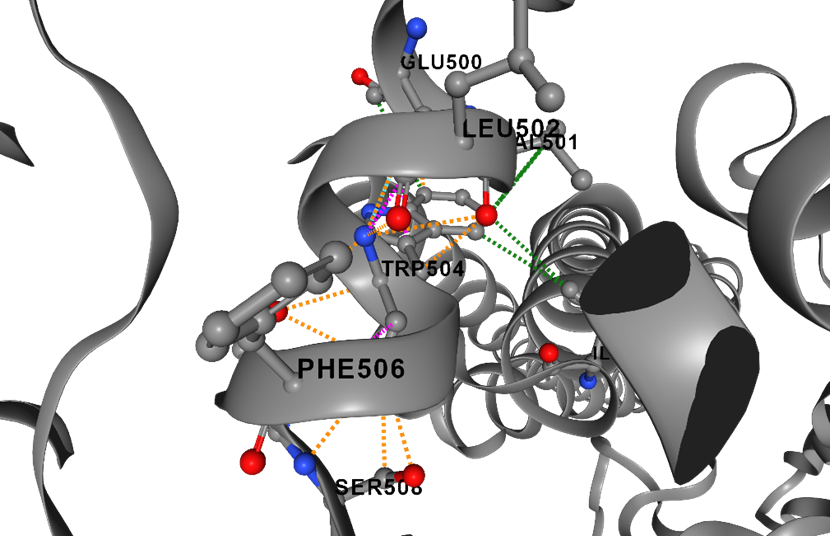

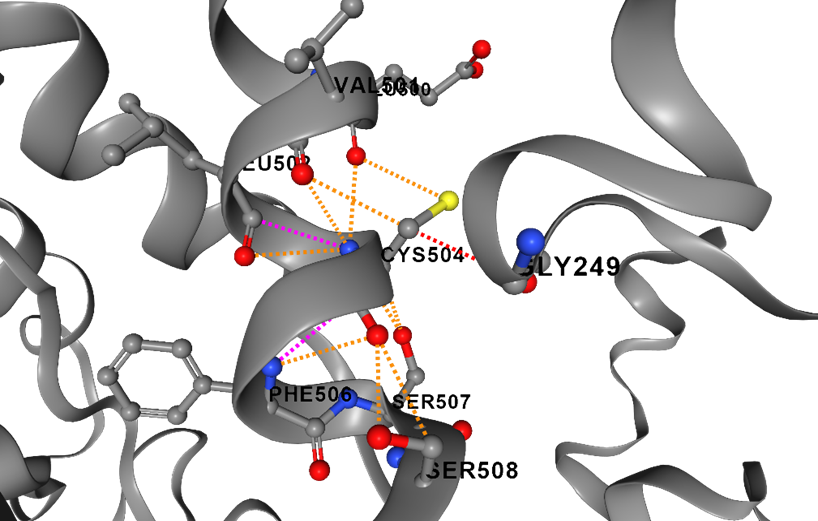


(A)W504C - wild type (B) W504C, mutant residue

|  |  |  |  |  |  |  |  |
| --- | --- | --- | --- | --- | --- | --- | --- |
| Hydrogen Bond | Hydrophobic | VDW | Clash | Ionic | Aromatic | Polar | Carbonyl |

Figure S6. Difference in ionic interactions between the wild-type (A) and mutant residues (B) in I648T.
